# Subthalamic nucleus stimulation attenuates motor seizures via modulating the nigral orexin pathway

**DOI:** 10.3389/fnins.2023.1157060

**Published:** 2023-05-05

**Authors:** Tao Xue, Shu Wang, Shujun Chen, Huizhi Wang, Chong Liu, Lin Shi, Yutong Bai, Chunkui Zhang, Chunlei Han, Jianguo Zhang

**Affiliations:** ^1^Department of Neurosurgery, Beijing Tiantan Hospital, Capital Medical University, Beijing, China; ^2^Department of Neurology, Beijing Tiantan Hospital, Capital Medical University, Beijing, China; ^3^Beijing Neurosurgical Institute, Capital Medical University, Beijing, China; ^4^Beijing Institute of Basic Medical Sciences, Beijing, China

**Keywords:** subthalamic nucleus, substantia nigra pars reticulata, deep brain stimulation, motor seizure, orexin

## Abstract

**Background:**

Focal motor seizures that originate in the motor region are a considerable challenge because of the high risk of permanent motor deficits after resection. Deep brain stimulation of the subthalamic nucleus (STN-DBS) is a potential treatment for motor epilepsy that may enhance the antiepileptic actions of the substantia nigra pars reticulata (SNr). Orexin and its receptors have a relationship with both STN-DBS and epilepsy. We aimed to investigate whether and how STN inputs to the SNr regulate seizures and the role of the orexin pathway in this process.

**Methods:**

A penicillin-induced motor epileptic model in adult male C57BL/6 J mice was established to evaluate the efficacy of STN-DBS in modulating seizure activities. Optogenetic and chemogenetic approaches were employed to regulate STN-SNr circuits. Selective orexin receptor type 1 and 2 antagonists were used to inhibit the orexin pathway.

**Results:**

First, we found that high-frequency ipsilateral or bilateral STN-DBS was effective in reducing seizure activity in the penicillin-induced motor epilepsy model. Second, inhibition of STN excitatory neurons and STN-SNr projections alleviates seizure activities, whereas their activation amplifies seizure activities. In addition, activation of the STN-SNr circuits also reversed the protective effect of STN-DBS on motor epilepsy. Finally, we observed that STN-DBS reduced the elevated expression of orexin and its receptors in the SNr during seizures and that using a combination of selective orexin receptor antagonists also reduced seizure activity.

**Conclusion:**

STN-DBS helps reduce motor seizure activity by inhibiting the STN-SNr circuit. Additionally, orexin receptor antagonists show potential in suppressing motor seizure activity and may be a promising therapeutic option in the future.

## Introduction

Epilepsy, one of the most prevalent neurological disorders, affects around 1% of the population (Devinsky et al., 2018). The pathological condition known as epilepsy is a circuit-level disease with hypersynchronous or excessive discharges and increased neuronal excitability as a result of an excitatory-inhibitory imbalance (Paz and Huguenard, 2015). Surgical resection is an effective therapeutic option for roughly 30% of individuals who continue to experience seizures despite receiving appropriate antiepileptic medications (Gomez-Alonso and Bellas-Lamas, 2015; Vakharia et al., 2018). Nonetheless, focal motor seizures originating in the motor region, particularly the primary motor cortex (M1), represent a considerable challenge due to the high risk of permanent motor deficits caused by ablation of the epileptic foci (Rosenow and Luders, 2001; Jobst and Cascino, 2015). As a result, the investigation of alternate therapy options for such individuals remains an active research area. Neuromodulatory approaches, such as deep brain stimulation (DBS), are hypothesized to normalize the abnormal brain activity and have been developed as an alternative treatment over the past several decades (Xue et al., 2022). The anterior nucleus of the thalamus (ANT), an important node in the Papez circuit, is currently the most commonly used stimulus target in the treatment of epilepsy (Fisher et al., 2010; Salanova et al., 2015). ANT-DBS demonstrated satisfactory control of temporal lobe seizures but less control of seizures arising from other regions (Fasano et al., 2021). Thus, for epileptic patients with motor seizures, especially for seizures originating from motor areas, it is necessary to explore new stimulation targets.

The subthalamic nucleus (STN), which plays an important role in the basal ganglia pathway, projects directly to the substantia nigra pars reticulata (SNr), which is a part of the well-known nigral regulatory system involved in epilepsy (Gale, 1992). In frontal lobe motor seizures, animal experiments have verified the activation of the indirect pathway, including STN, SNr, and their upstream and downstream nuclei (Brodovskaya et al., 2021). STN-DBS has already been widely used in movement disorders, such as Parkinson’s disease (Weaver et al., 2009), and is thought to enhance the antiepileptic actions of SNr (Salanova, 2018). Despite several clinical case reports and pilot studies that have identified its potential role in the treatment of motor seizures (Benabid et al., 2002; Chabardes et al., 2002; Handforth et al., 2006; Lee et al., 2006; Ren et al., 2020), the exact neural circuit mechanism of STN-DBS in controlling seizures remains unclear. The excitatory hypothalamic peptide orexin (also known as hypocretin), which has two subtypes named orexin A (OA) and orexin B (OB), is secreted by a group of neurons located in the hypothalamus close to the STN that project to both STN and SNr (Peyron et al., 1998). Furthermore, STN and SNr have two types of orexin receptors, OX1R and OX2R, which are co-localized in these two regions (Korotkova et al., 2002; Li et al., 2019). Endogenous orexin A/B maintains the physiological discharge of STN glutamatergic neurons and SNr GABAergic neurons (Korotkova et al., 2002; Sheng et al., 2018). In addition, exogenous extra orexin A/B was found to increase penicillin-induced epileptic activity (Kortunay et al., 2012), and orexin receptor antagonisms were observed to reduce seizures in multiple epilepsy models (Zhu et al., 2015; Roundtree et al., 2016; Kordi Jaz et al., 2017). Meanwhile, DBS can affect the expression of orexin receptors (Dong et al., 2021). Therefore, we systematically investigated whether and how STN inputs to SNr regulate seizures and the role that the orexin pathway plays in this process using a mouse model in which epilepsy originated from M1. Identifying the neural circuitry and molecular mechanism responsible for motor seizures could result in the development of more precise therapeutic approaches to control seizures.

## Materials and methods

### Animals

In the study, 238 male C57BL/6 J mice, aged 6–8 weeks, were used. The mice were group-housed in cages containing 4–6 mice and were kept in a controlled environment with a 12-h light/dark cycle and a temperature of 20–23°C. The mice had free access to food and water and were cared for in accordance with the National Institute of Health Guide for the Care and Use of Laboratory Animals. All procedures involving the mice were approved by the Animal Advisory Committee of the Beijing Tiantan Hospital and conformed to the Animal Research: Reporting *In Vivo* Experiments (ARRIVE) guidelines. Information about mice mortality and exclusion can be found in Supplementary Table 1.

### Stereotactic surgery

Mice were anesthetized using sodium pentobarbital (50 mg/kg, i.p., Sigma-Aldrich) and had their heads fixed in a stereotaxic apparatus (68,044, RWD Instruments). To ensure that the mice remained pain-free during the procedure, additional doses of sodium pentobarbital were administered if a pain response was observed upon paw pinch. The body temperature of the anesthetized mice was maintained at 37°C using a heating pad. An incision was made on the head to expose the skull, and burr holes were stereotactically made on the skull after removing the pericranium.

Microinjections were administered using a gauge needle and an Ultra Micro Pump (R-480, RWD Instruments) for viral delivery, with the coordinates for the left STN, left SNr, and left M1 being −2.0 mm AP, +1.6 mm ML, −4.6 mm V; −3.4 mm AP, +1.5 mm ML, −4.4 mm V; and + 2.1 mm AP, +2 mm ML, −1.7 mm V from Bregma, respectively, based on the mouse brain atlas (Paxinos and Franklin, 2004). The viruses were infused over a period of 5–10 min, and the syringe was left in place until 10 min after the end of the infusion to allow for diffusion. Adeno-associated viruses (AAVs) were injected at a total volume of 0.1–0.5 μl, based on the size of the brain region, and approximately 3 weeks were allowed for maximal viral expression.

After 3 weeks of viral delivery, bipolar stimulation electrodes (CBBRF50, FHC), optical fibers (ULC-589-200-0.73-4.0, Newdoon Inc), or single-guide cannulas (62,001, RWD Instruments) were implanted 300 μm above the center of the M1, STN, or SNr, based on the specific experiment, using stereotaxic coordinates. These were fixed to the skull using dental cement, and three anchoring skull screws (diameter of 1 mm) were placed in the skull also for EEG recording, two of which were placed over the left neocortex (first electrode, −1.00 mm AP, +1.5 mm ML; second electrode, +3.00 mm AP, +1.5 mm ML), one of which was placed over the cerebellum to serve as the reference electrode. The implantation site and viral expression in the mice were verified after behavioral testing, and only mice with correct implantation location and viral expression were included in the analysis. Throughout the surgical procedures, the mice were kept on a heating pad and were returned to their home cages after post-surgery recovery.

### Seizure induction

After 1 week of recovery from stereotactic surgery, penicillin was administered to freely moving mice through a guide cannula implanted in the M1 region using an Ultra Micro Pump (KDS LEGATO 130, RWD Instruments). The infusion rate was set at 0.1 μl/min, with a total injection of 1 μl of penicillin (200 IU/μL, P105489, Aladdin). EEG recordings were obtained beginning 10 min prior to the penicillin injection and continued for 180 min. Thirty minutes before the penicillin injection, the mice in the different groups received either a saline injection (i.p.) or CNO (1 mg/kg, i.p.), depending on the specific experiment being conducted. The severity of behavioral seizures was evaluated using Racine’s criteria (Racine, 1972), which included the following: (1) facial movement, (2) head nodding, (3) unilateral forelimb clonus, (4) bilateral forelimb clonus and rearing, and (5) rearing and falling. Seizure stages 1–3 was classified as focal seizures (FSs), and stages 4–5 were classified as general seizures (GSs). The number of FSs and GSs were recorded by an investigator who was unaware of the group assignments during a 3-h observation period.

### DBS and photostimulation

The DBS electrodes were connected to a stimulator (Master-8 Programmable Stimulator, AMPI, Jerusalem, Israel), which delivered electrical pulses (pulse width = 60 μs, intensity = 100 μA) at specific frequencies based on the experimental design. The electrodes of the sham group were not connected to the stimulator, and therefore, the animals in this group did not receive any stimulation.

Blue (465 nm, 30 Hz, 10 ms, 10 mW) or yellow (589 nm, continuous, 10 mW) laser light was delivered using an optical stimulation system (IOS-465/589, RWD Instruments). For the negative control group, the parameters of the laser were adjusted to be the same as those used in the experimental group. The power of the laser was adjusted to approximately 10 mW, as measured using a power meter (Thorlabs).

### EEG recordings and fiber photometry

EEG activity was recorded using a data acquisition system (PowerLab, AD Instruments). The biological signals from the electrodes were amplified and filtered (bandpass of 0.1–50 Hz) using BioAmp amplifiers (AD Instruments). The EEG signal was digitized at a sampling rate of 1,024 Hz and displayed and stored on a personal computer. The frequency and amplitude of epileptiform EEG activity were analyzed offline using LabChart software (AD Instruments) (Yildirim et al., 2010). Only spikes with amplitudes greater than three times the baseline activity were included in the analysis. The EEG power spectrum was analyzed offline using the basal activity recorded prior to the penicillin injection. Only the 0.5–50 Hz range of all spectra was used for further analysis, with faster frequency bands being cropped. EEG power was calculated in the following frequency bands: delta (0.5–4 Hz), theta (4–8 Hz), alpha (8–13 Hz), beta (13–30 Hz), and gamma (30–50 Hz). Examples of EEG recordings for different periods of epileptic activity can be found in Supplementary Figure S1A.

Fiber photometry of calcium signals was initiated 1 week after the implantation surgery. GCaMP fluorescence was collected using a fiber photometry system (inper) and analyzed using a supporting data processing software (Inper Data Process, inper). The data were segmented according to individual trials, and the values of fluorescence change (ΔF/F) were calculated as (F − F0)/F0. Heatmaps or average plots were used to present the ΔF/F values. Examples of calcium signals after triple injection of AAVs in three brain regions: M1, STN and SNr during seizures can be found in Supplementary Figure S1B.

### Immunofluorescence labeling

Mice were anesthetized and transcardially perfused with 0.9% NaCl and then with 4% paraformaldehyde (PFA) in phosphate buffer solution (PBS). Their brains were dissected, fixed in 4% PFA for 6 h at 4°C, and transferred to solutions of 20 and 30% sucrose in PBS for 36–48 h. The brains were coronally sectioned into 40-μm slices using a freezing microtome (Leica, CM1950), and the slices were collected for immunostaining. The slices were pre-incubated in a solution of 3% bovine serum albumin (w/v) and 0.5% Triton X-100 (v/v) for 2 h at room temperature, followed by overnight incubation at 4°C with the appropriate primary antibodies (listed in Supplementary Table S2). Among them, the antibody of c-Fos was used to determine the enhancement neural activity induced by penicillin and the antibodies of OX1R, OX2R were used to detect the expression and colocalization in SNr. They were then incubated with fluorescent secondary antibodies (Alexa fluor 488-conjugated goat anti-rabbit [A11008]; Alexa fluor 546-conjugated donkey anti-rabbit [A10040]; Invitrogen; all 1:2000) for 2 h at room temperature. The primary and secondary antibodies were diluted in PBS containing 3% bovine serum albumin (w/v) and 0.3% Triton X-100. The fluorescent signals were examined and photographed using an A1R laser-scanning confocal microscope (Nikon A1R, Japan) with magnification = 10× and numerical aperture = 0.45. The relative fluorescence intensity was analyzed using ImageJ software by observers who were blinded to the experimental groups.

### Western blot analysis

Briefly, brain tissues containing SNr were homogenized in 1% sodium dodecyl sulfate (SDS). Protein aliquots of 20 μl from each sample were separated by 8% SDS-polyacrylamide gel electrophoresis and transferred to a polyvinylidene fluoride membrane. The membrane was blocked with 3% non-fat milk in PBS at room temperature for 2 h and then incubated with the primary antibodies [rabbit-anti-OX1R or rabbit-anti-OX2R (details in Supplementary Table S2) or mouse-anti-GAPDH (Invitrogen, PA1-988, 1:1000)] overnight at 4°C. After being washed three times, the membrane was incubated with HRP (anti-mouse: sc-2004; anti-rabbit: sc-2005, Santa Cruz Biotechnology) at room temperature, and the protein bands were visualized using the ECL system (Tanon Inc., 5,200). The gray scale of the bands was quantified using ImageJ software.

### Elisa

The acquired supernatants of SNr were collected and stored at −80°C until use. All operations of ELISA for OA (EM0453, Wuhan Fine Biotech Co.) and OB (CX4453, Shanghai Chuangxiang Co.) were conducted according to the manufacturer’s instructions.

### Viral vectors and drugs

rAAV-CaMKIIα-mCherry (titre: 1.2 × 10^13^ v.g.ml^−1^), rAAV/retro-hSyn-eYFP (titre: 1.3× 10^13^ v.g.ml^−1^), rAAV-CaMKIIa-GCaMp6m (titre: 1.1× 10^13^ v.g.ml^−1^), rAAV-CaMKIIα-hChR2 (H134R)-eYFP (titre: 5.8 × 10^12^ v.g.ml^−1^), rAAV-CaMKIIα-eNpHR-eYFP (titre: 5.2 × 10^12^ v.g.ml^−1^), rAAV-EF1α-DIO-hM3D (Gq)-mCherry (titre: 1.7 × 10^13^ v.g.ml^−1^), rAAV-EF1α-DIO-hM4D(Gi)-mCherry (titre: 2.2 × 10^13^ v.g.ml^−1^), and rAAV/retro-hSyn-CRE (titre: 1.8 × 10^13^ v.g.ml^−1^) were purchased from BrainVTA Co., Ltd. (Wuhan, China). All viral vectors were aliquoted and stored at −80°C until use.

The selective antagonists for OX1R and OX2R, named SB-334867 (10 μM, item no. 19145) and JNJ-10397049 (10 μM, item no. 14139), were purchased from Cayman Chemical Co. (Ann Arbor, MI, United States) and stored at −20°C until use.

### Statistics

Data are presented as means ± SD. The number of experimental replicates (n) is indicated in the figures and refers to the number of experimental subjects that were independently treated in each experimental condition. Statistical comparisons were conducted using Prism (version 8.0) with the appropriate methods. No statistical methods were applied to pre-determine the sample size or to randomize the groups. All analyses were two-tailed, and a value of *p* <0.05 was considered statistically significant. A detailed statistical description of all figures can be found in Supplementary Table S3.

## Results

### Ipsilateral/bilateral high-frequency (130 Hz) STN-DBS alleviates seizures in motor epilepsy

**Figure 1:**
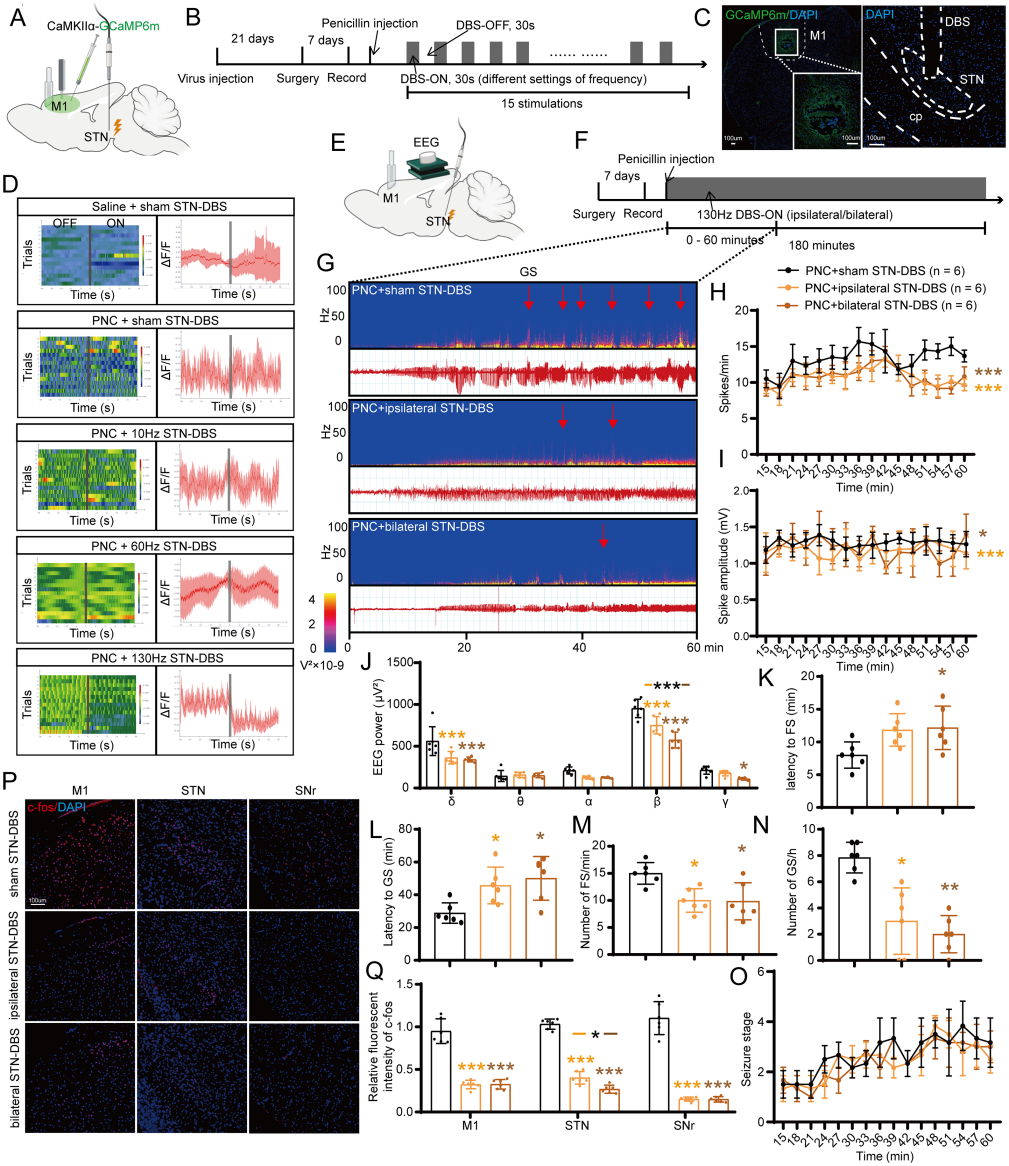
Effects of deep brain stimulation of the subthalamic nucleus (STN-DBS) on motor epilepsy. **(A,B)** Scheme and time course of the experiment for the efficacy of different frequencies of STN-DBS in motor epileptic mice. **(C)** Representative images of the primary motor cortex (M1) and STN, confirming the expression of CaMKIIα-GCaMP6m and location of the electrode. Scale bar = 100 μm. Left, M1; Right, STN. **(D)** Fiber photometry of M1 excitatory neural dynamics during motor seizures in CaMKIIα-GCaMP6m^M1^ mice according to different groups. Left column, heatmap illustration of calcium signals. Color scale indicates ΔF/F, and warmer colors indicate higher fluorescence signals; Right column, peri-event plots of the average calcium signals corresponding to the heatmaps. Red lines indicate mean, and shaded areas indicate SD. The gray lines represent electrical stimulation. **(E,F)** Scheme and time course of the experiment for the efficacy of ipsilateral and bilateral high-frequency (130 Hz) STN-DBS in motor epileptic mice. **(G)** Representative EEG spectra power and raw EEG; red arrowheads indicate generalized seizure (GS) onset. Effects of ipsilateral and bilateral high-frequency (130 Hz) STN-DBS on **(H)** spike frequency, **(I)** spike amplitude, **(J)** spectral power density, **(K)** latency to FS, **(L)** latency to GS, **(M)** number of FS, **(N)** number of GS, and **(O)** development of seizure stage. **(P)** Immunofluorescence analysis was performed using antibodies against c-Fos (red) in brain sections of M1, STN, and substantia nigra pars reticulata (SNr). Nuclei were fluorescently labeled with DAPI (blue). Scale bar = 100 μm. **(Q)** Relative fluorescence intensity of c-Fos in M1, STN, and SNr. *p < 0.05, **p < 0.01, ***p < 0.001. Colored asterisk indicates the comparison of the corresponding group and the penicillin + sham STN-DBS group; black asterisk with two different colored horizontal lines to the left and right represents comparison of the corresponding two groups. Data are presented as means ± SD. Detailed statistical methods and data are provided in Supplementary materials.

To examine the effects of different frequencies of STN-DBS on seizure control in motor epilepsy, we recorded the calcium signals of M1 excitatory neurons using fiber photometry in freely moving epileptic mice with STN-DBS (ipsilateral stimulation, 10/60/130 Hz, 100 μA, 60 μs, 30-s on–off cycle; Figures 1A,B). Following stereotaxic infusion of the AAVs into M1, the calcium indicator GCaMP6m was efficiently expressed in M1 neurons (Figure 1C). Then, we implanted an optical fiber into the M1 for recordings of GCaMP fluorescence changes and an electrode into the STN to induce electrical stimulation (Figure 1C; Supplementary Figure S1C). We observed an increased calcium response in ipsilateral M1 after injection of penicillin and sham. Low-frequency (10 Hz) and moderate-frequency (60 Hz) stimulation of STN did not attenuate the calcium signal of seizures, while high-frequency (130 Hz) STN-DBS alleviated the seizures (Figure 1D). Therefore, the frequency setting of 130 Hz was used for subsequent experiments.

Next, we aimed to assess whether ipsilateral and bilateral high-frequency STN-DBS had different effects on seizure improvement. Continuous 130-Hz stimulation was used to identify the efficacy of ipsilateral/bilateral STN-DBS for motor seizure control (Figures 1E,F). A typical EEG and the corresponding power spectrum are shown in Figure 1G. EEG analysis demonstrated that both ipsilateral and bilateral stimulation significantly decreased the spike frequency (Figure 1H), amplitude (Figure 1I), and power spectral density (Figure 1J) of motor seizures. In addition, ipsilateral STN-DBS significantly prolonged the latency to GS, whereas bilateral stimulation significantly prolonged the onset of both FS and GS (Figures 1K,L). Meanwhile, ipsilateral/bilateral STN-DBS distinctly reduced the number of FSs and GSs (Figures 1M,N; Supplementary Figure S1D) but failed to prevent the development of the seizure stages (Figure 1O). Furthermore, the neurons in M1, STN, and SNr were activated after penicillin injection with c-Fos expression (Supplementary Figure S1E). Ipsilateral/bilateral STN-DBS significantly reduced the fluorescent intensity of c-Fos (Figures 1P,Q), suggesting that STN-DBS reversed the hyperexcitability of multiple brain regions affected by the motor epilepsy network. The above results suggest that both ipsilateral and bilateral high-frequency STN-DBS have similar effects in improving seizures in the mouse motor epilepsy model. Therefore, after various considerations of efficacy, cost, and convenience, ipsilateral 130-Hz STN-DBS was considered to be effective and was used in subsequent relevant experiments.

### Activation of STN excitatory neurons amplifies seizure activities

**Figure 2:**
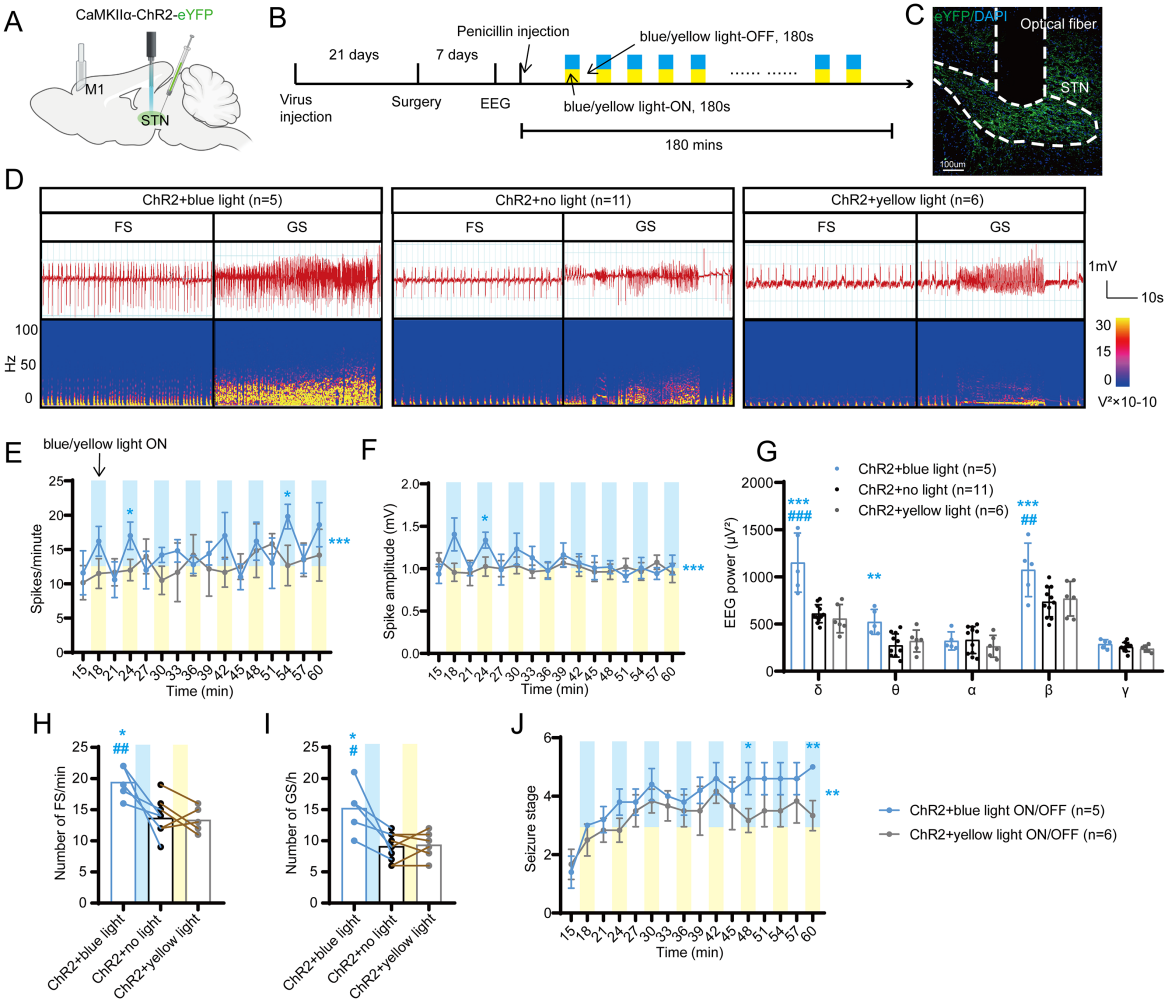
Optogenetic activation of the subthalamic nucleus (STN) excitatory neurons amplifies seizure activities in motor epilepsy. **(A,B)** Scheme and time course of the experiment for viral injection and photostimulation of motor epileptic mice. **(C)** Representative images of the STN, confirming the expression of CaMKIIα-ChR2-eYFP and location of the optic fiber. Scale bar = 100 μm. **(D)** Representative EEGs and corresponding EEG spectra power for focal seizure (FS) and generalized seizure (GS). Effects of optogenetic activation of the STN excitatory neurons on **(E)** spike frequency, **(F)** spike amplitude, **(G)** spectral power density, **(H)** number of FS, **(I)** number of GS, and **(J)** development of seizure stage. For panels **(E,F,J)**, *p < 0.05, **p < 0.01, ***p < 0.001. Colored asterisk located at the right side of the curve indicates comparison through the entire period; colored asterisk located above the curve indicates comparison at the specific time point. The half-blue and half-yellow rectangles indicate photostimulation according to the groups. For panels **(G–I)**, */#*p* < 0.05, **/##*p* < 0.01, ***/###*p* < 0.001. Asterisk indicates comparison with the ChR2 + no light group; pound symbol indicates comparison with the ChR2 + yellow light group. Data are presented as means ± SD. Detailed statistical methods and data are provided in Supplementary materials.

To investigate the role of STN excitatory neurons (most of them being glutamatergic neurons) in seizures of motor epilepsy, we used an optogenetic approach to selectively stimulate Channelrhodopsin 2 (ChR2)-expressing excitatory neurons in the STN of CamKIIα-ChR2-eYFP mice (Figures 2A,B). Histological data confirmed the presence of eYFP-expressing neurons and an inserted optical fiber in the STN (Figure 2C). Representative EEG and power spectrum confirmed that blue-light stimulation (465 nm, 30 Hz, 10 ms, 10 mW, 180-s on–off cycle) amplified seizure activities in six mice, suggesting that activation of STN excitatory neurons can deteriorate seizures originating from M1. Yellow-light stimulation (589 nm, 30 Hz, 10 ms, 10 mW, 180-s on–off cycle), serving as the control stimulation, was associated with similar seizure activities as no-light stimulation (Figure 2D). Statistics analysis of EEG also revealed that mice in the ChR2 + blue light ON/OFF group (*n* = 5) had higher spike frequency (Figure 2E) and amplitude (Figure 2F) than those in the ChR2 + yellow light ON/OFF group; the effect was more pronounced especially during the initial few stimulations (within 30 min after injection). In addition, blue-light stimulation enhanced the spectral density and increased the number of FSs and GSs, while yellow-light stimulation had no effect, compared to no light (Figures 2G–I). Finally, the 180-s ON/OFF stimulation with blue light could significantly accelerate the seizure stages compared to yellow light (Figure 2J). Thus, the above results illustrate that driving STN excitatory neurons amplify seizure activities in the penicillin motor epilepsy model.

### Inhibition of STN excitatory neurons attenuates seizure activities

**Figure 3:**
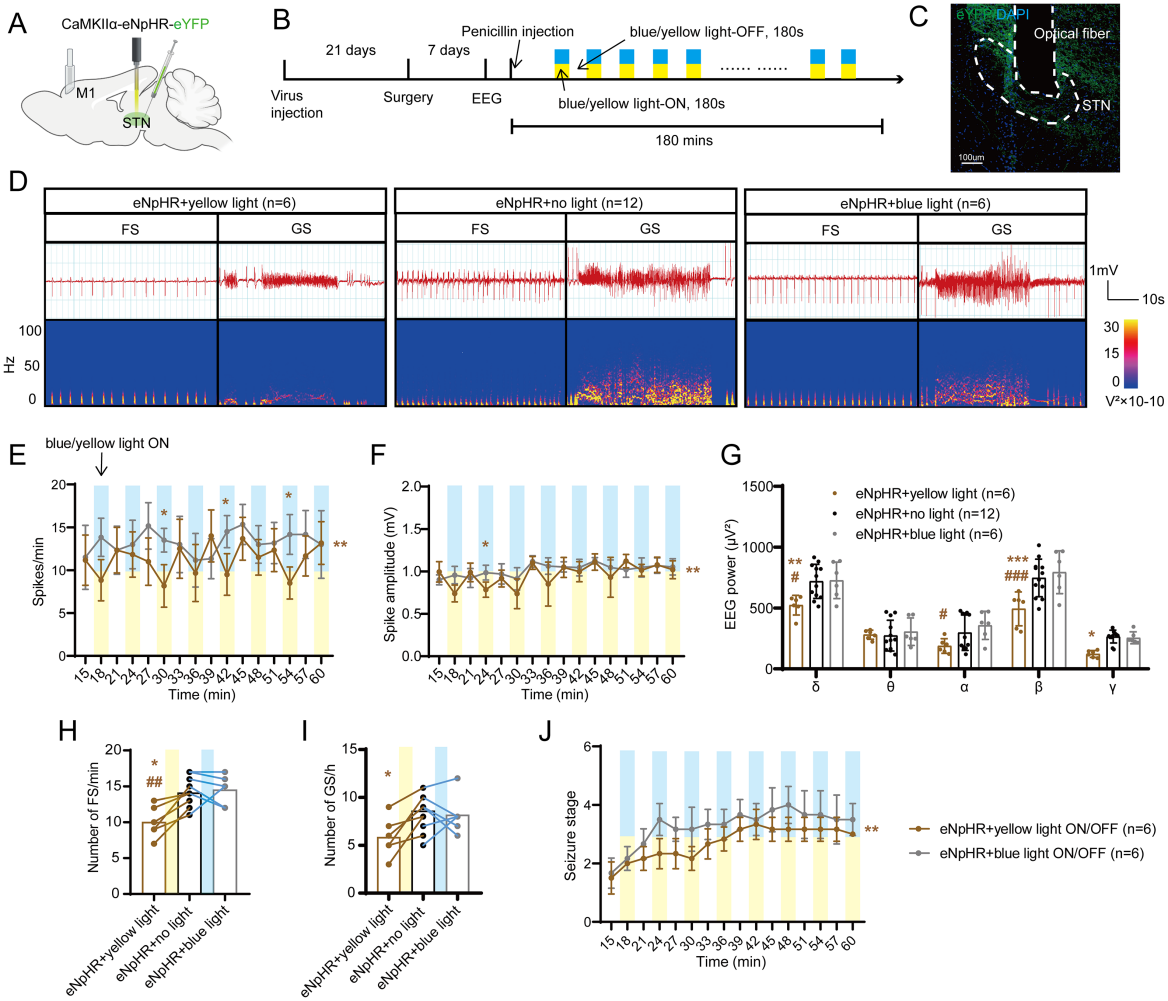
Optogenetic inhibition of the subthalamic nucleus (STN) excitatory neurons amplifies seizure activities in motor epilepsy. **(A,B)** Scheme and time course of the experiment for viral injection and photostimulation of motor epileptic mice. **(C)** Representative images of the STN, confirming the expression of CaMKIIα-eNpHR-eYFP and location of the optic fiber. Scale bar = 100 μm. **(D)** Representative EEGs and corresponding EEG spectra power for focal seizure (FS) and generalized seizure (GS). Effects of optogenetic inhibition of the STN excitatory neurons on **(E)** spike frequency, **(F)** spike amplitude, **(G)** spectral power density, **(H)** number of FS, **(I)** number of GS, and **(J)** development of seizure stage. For panels **(E,F,J),** *p < 0.05, **p < 0.01. Colored asterisk located at the right side of curve indicates comparison with the entire period; colored asterisk located above the curve indicates comparison at the specific time point. The half-blue and half-yellow rectangles represent photostimulation according to the group. For panels **(G–I),** */#*p* < 0.05, **/##*p* < 0.01, ***/###*p* < 0.001. Asterisk indicates comparison with the eNpHR + no light group; pound symbol indicates comparison with the eNpHR + blue light group. Data are presented as means ± SD. Detailed statistical methods and data are provided in Supplementary materials.

To further test whether STN excitatory neurons are required for seizures of motor epilepsy, we introduced eNpHR into the STN of mice to selectively photo-inhibit STN excitatory neurons (Figures 3A,B). Histological data confirmed the expression of eNpHR-eYFP and the location of the fiber in the STN (Figure 3C). Figure 3D shows the typical EEG and power spectrum, indicating that yellow-light stimulation (589 nm, continuous, 10 mW, 180-s on–off cycle) attenuated seizure activities and blue-light stimulation (465 nm, continuous, 10 mW, 180-s on–off cycle) exerted similar effects as no light. Meanwhile, EEG analysis demonstrated that mice in the eNpHR + yellow light ON/OFF group (*n* = 6) had fewer spikes (Figure 3E) and lower spike amplitudes (Figure 3F) than those in the eNpHR + blue light ON/OFF group; yellow light significantly reduced the EEG power compared to blue light and no light in δ and β bands (Figure 3G). In addition, yellow light significantly reduced the numbers of FSs compared to no light and blue light, and significantly decreased the numbers of GSs compared to no light (Figures 3H,I). Finally, yellow light ON/OFF circulation slowed down the development of the seizure stages compared to blue light (Figure 3J). According to the evidence presented above, STN excitatory neurons bidirectionally regulate the magnitude of seizures in the motor epilepsy model. Activation of STN neurons amplifies seizures, while inhibition of STN neurons alleviates seizures.

### STN-SNr circuit bidirectionally regulates seizures

Next, we aimed to test how STN neurons are involved in seizure regulation of motor epilepsy. Previous studies have found that frontal epilepsy originating from the supplementary motor area is more likely to activate dopamine D2 receptor-expressing neurons in the indirect pathway (Brodovskaya et al., 2021). Moreover, in the basal ganglia circuit, the downstream nuclear outputs of the STN are mainly through SNr and globus pallidus internus (GPi). Additionally, SNr, instead of GPi, has also been found to be closely related to a type of epilepsy. Therefore, we mainly explored whether the STN-SNr circuit plays an important role in motor epilepsy. The classic STN-SNr projections were briefly verified by anterograde and retrograde tracer AAVs (Supplementary Figures S1F,G).

**Figure 4:**
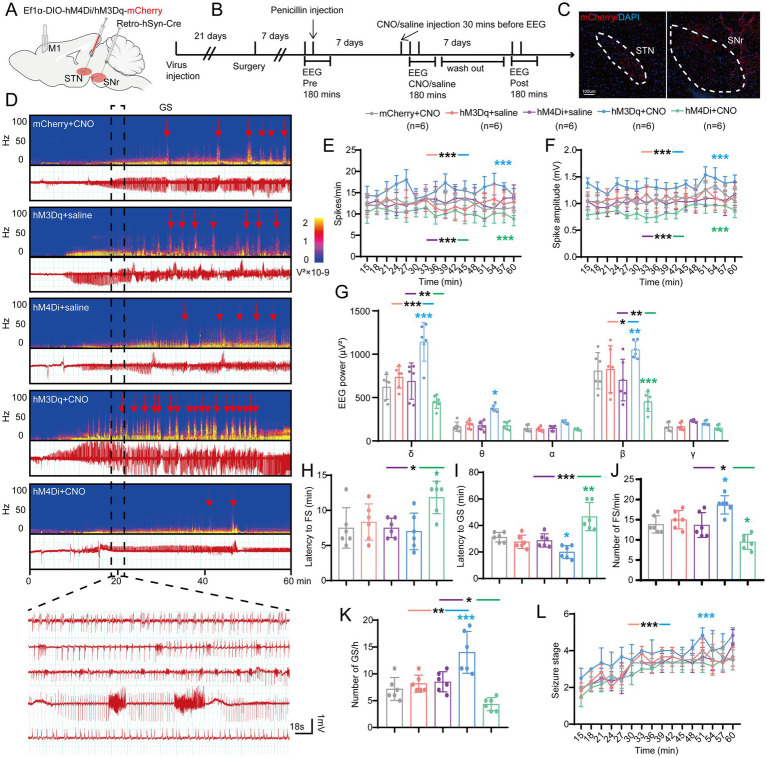
Effects of chemogenetic regulation of the subthalamic nucleus-substantia nigra pars reticulata (STN-SNr) circuit on motor epilepsy. **(A,B)** Scheme and time course of experiment. **(C)** Representative images of the STN and SNr, confirming the expression of virus. Scale bar = 100 μm. Left, STN; Right, SNr. **(D)** Representative EEG spectra power and raw EEG; below are enlarged raw EEG segments corresponding to the dotted boxes; red arrowheads indicate generalized seizure (GS) onset. Effects of chemogenetic regulation of the STN-SNr circuit on **(E)** spike frequency, **(F)** spike amplitude, **(G)** spectral power density, **(H)** latency to FS, **(I)** latency to GS, **(J)** number of FS, **(K)** number of FS, and **(L)** development of seizure stage. *p < 0.05, **p < 0.01, ***p < 0.001. Colored asterisk indicates comparison of the corresponding group and the mCherry + CNO group; black asterisk with two different colored horizontal lines to the left and right indicates comparison of the corresponding two groups. Data are presented as means ± SD. Detailed statistical methods and data are provided in Supplementary materials.

Initially, we adopted a chemogenetic method to observe whether enhancing or suppressing the activity of STN-SNr projections can alleviate motor seizures. AAV-Ef1α-DIO-hM4Di/hM3Dq/(empty)-mCherry and AAV2-retro-hSyn-Cre were injected into the STN and SNr, respectively (Figures 4A,B). The first virus expressed hM4Di/hM3Dq, two designed G-protein-coupled receptors sensitive to the metabolite of clozapine, clozapine-N-oxide (CNO), and was usually employed to suppress/enhance neuronal activity (Urban and Roth, 2015). Histological data confirmed the mCherry-expressing neurons in the STN and SNr (Figure 4C). STN-SNr hM4Di/hM3Dq-mCherry mice received CNO (i.p. 1 mg/kg in saline vehicle) half an hour before penicillin injection (200 IU/μL, 1.0 μl) *via* the implanted cannula guide into the M1. STN-SNr hM4Di/hM3Dq-mCherry mice treated with saline or STN-SNr mCherry mice treated with CNO served as controls. The representative graphs of EEG and power spectrum are shown in Figure 4D. Chemogenetic suppression of STN-SNr projections substantially reduced spike frequency (Figure 4E), spike amplitude (Figure 4F), and EEG density (Figure 4G), prolonged latency to FSs (Figure 4H) and GSs (Figure 4I), lowered the number of FSs and GSs (Figures 4J,K) but failed to retard seizure progression (Figure 4L). Conversely, activation of the STN-SNr circuit significantly increased spike frequency (Figure 4E), spike amplitude (Figure 4F), and EEG density (Figure 4G) and shortened the latency to GSs (Figure 4I) but not FSs (Figure 4H). It also increased the number of FSs, GSs (Figures 4J,K; Supplementary Figure S3A) and accelerated the development of the seizure stages (Figure 4L). In addition, on the 7th day before and after the use of CNO/saline, tests without any drugs were conducted to further confirm the effect of chemogenetic inhibition/activation on motor seizure activities *via* self-comparison of the data before, at, and after the administration of CNO in each group. The results showed that mice in the mCherry + CNO group had similar seizure numbers and latency to FS at pre, CNO, and post time points (Supplementary Figures S2A–D). STN-SNr hM3Dq mice had more frequent seizures and shorter latency to FSs and GSs at the time of injection of CNO (Supplementary Figures S2E–H). Moreover, two mice died after CNO injection (Supplementary Table 1). HM4Di exhibited the opposite effects (Supplementary Figures S2I–L).

**Figure 5:**
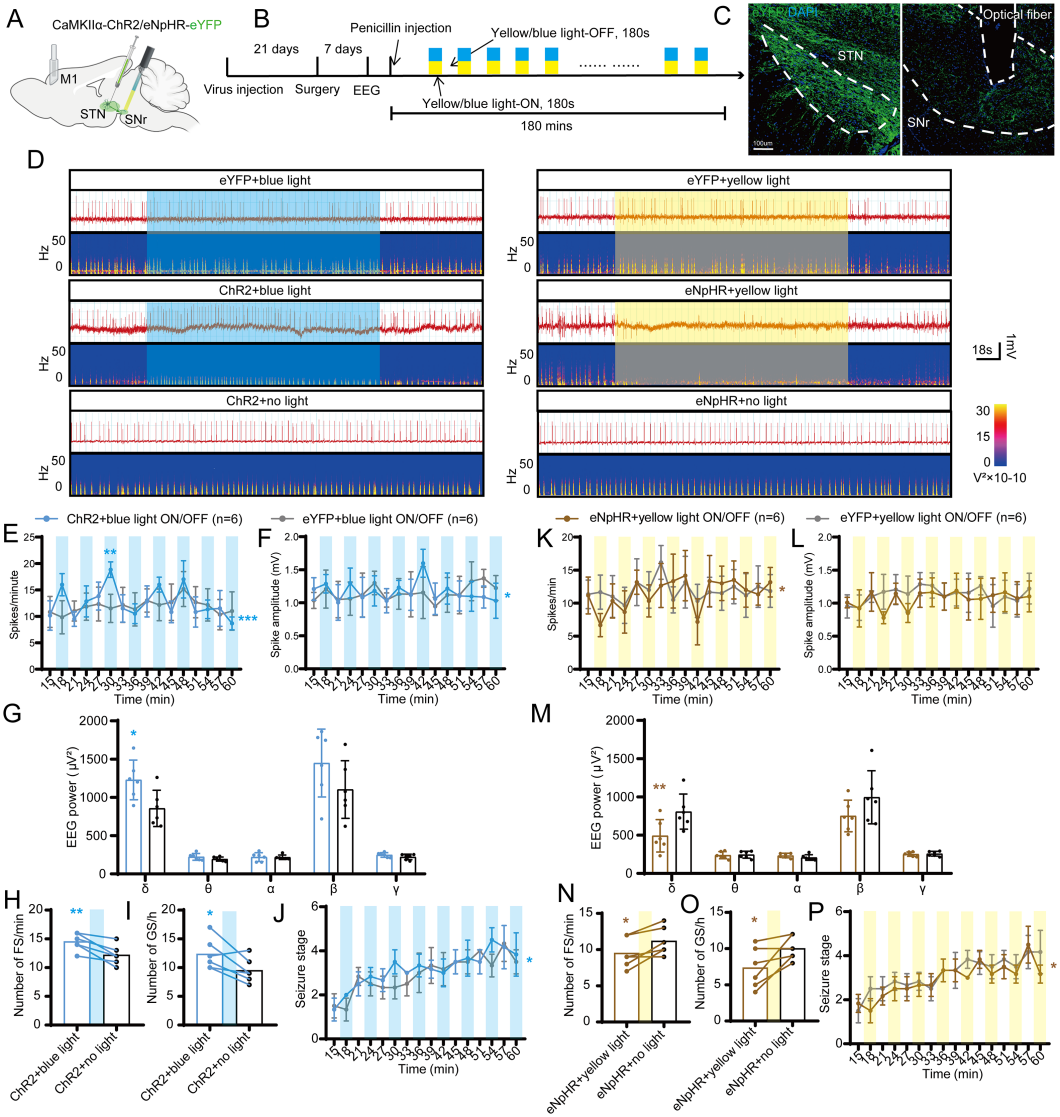
Effects of optogenetic regulation of the subthalamic nucleus-substantia nigra pars reticulata (STN-SNr) circuit on motor epilepsy. **(A,B)** Scheme and time course of experiment. **(C)** Representative images of the STN and SNr, confirming the expression of virus and location of the optic fiber. Scale bar = 100 μm. Left, STN; Right, SNr. **(D)** Representative EEGs and corresponding EEG spectra power for focal seizure (FS). Effects of optogenetic activation of the STN-SNr circuit on **(E)** spike frequency, **(F)** spike amplitude, **(G)** spectral power density, **(H)** number of FS, **(I)** number of generalized seizure (GS), and **(J)** development of seizure stage. Effects of optogenetic inhibition of the STN-SNr circuit on **(K)** spike frequency, **(L)** spike amplitude, **(M)** spectral power density, **(N)** number of FS, **(O)** number of generalized seizure (GS), and **(P)** development of seizure stage. *p < 0.05, **p < 0.01, ***p < 0.001. Colored rectangles represent photostimulation. Data are presented as means ± SD. Detailed statistical methods and data are provided in Supplementary materials.

Second, to further verify whether photo-activation or photo-inhibition of STN-SNr projections can regulate seizure activity of motor epilepsy, we injected CamKIIα-ChR2/eNpHR/(empty)-eYFP into the STN and implanted an optic fiber into the SNr delivering intermittent blue/yellow-light stimulation to activate or inhibit the STN inputs to SNr (Figures 5A,B). Histological data confirmed the expression of eYFP in the STN and the location of the fiber in the SNr (Figure 5C). STN-SNr eYFP mice treated with blue/yellow-light or STN-SNr ChR2/eNpHR mice treated with no light served as controls. The typical EEG with power spectra found that ChR2 + blue-light stimulation (465 nm, 30 Hz, 10 ms, 10 mW, 180-s on–off cycle) deteriorated seizure activities, while eNpHR+ yellow-light stimulation (589 nm, continuous, 10 mW, 180-s on–off cycle) improved seizure activities (Figure 5D). Photo-activation of STN-SNr circuit increased spike frequency (Figure 5E), spike amplitude (Figure 5F), power spectral density (Figure 5G), and number of FSs (Figure 5H) and GSs (Figure 5I) and accelerated seizure progression (Figure 5J). Photo-inhibition exerted opposite effects, except that it did not significantly lower spike amplitude (Figures 5K–P). The aforementioned data show that STN-SNr projections modulate seizure severity in a bidirectional manner in motor epilepsy models, indicating that the anti−/pro-epileptic action was mediated by STN-SNr direct projections and not by the passing fibers.

### STN-DBS alleviates seizures by inhibiting the STN-SNr circuit

**Figure 6:**
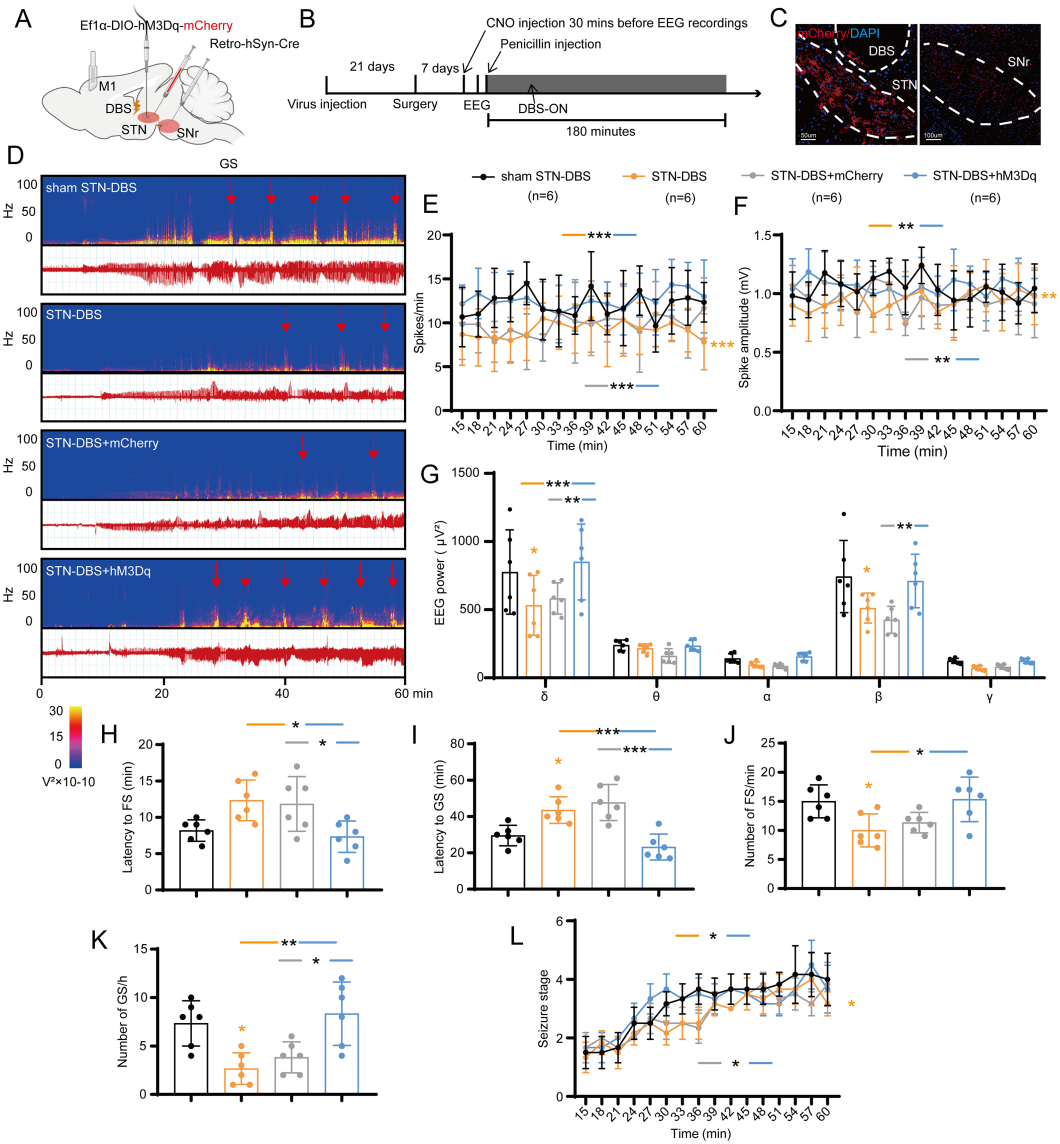
Deep brain stimulation of the subthalamic nucleus (STN-DBS) alleviates seizures by inhibiting the STN-substantia nigra pars reticulata (STN-SNr) circuit. **(A,B)** Scheme and time course of experiment. **(C)** Representative images of the STN and SNr, confirming the expression of the virus and the location of the electrode. Left, STN, Scale bar = 50 μm; Right, SNr, Scale bar = 100 μm. **(D)** Representative EEG spectra power and raw EEG; red arrowheads indicate generalized seizure (GS) onset. Effects of ipsilateral high-frequency (130 Hz) STN-DBS with chemogenetic activation of the STN-SNr circuit on **(E)** spike frequency, **(F)** spike amplitude, **(G)** spectral power density, **(H)** latency to FS, **(I)** latency to GS, **(J)** number of focal seizure (FS), **(K)** number of GS, and **(L)** development of seizure stage. *p < 0.05, **p < 0.01, ***p < 0.001. Colored asterisk indicates comparison of the corresponding group and the sham STN-DBS group; black asterisk with two different colored horizontal lines to the left and right indicates comparison of the corresponding two groups. Data are presented as means ± SD. Detailed statistical methods and data are provided in Supplementary materials.

According to the above-mentioned results, we found that high-frequency STN electrical stimulation can exert a similar effect on the inhibition of STN excitatory neurons or STN-SNr circuit. Therefore, we speculated that STN-DBS can improve epileptic activity in mice by inhibiting STN-SNr projections. To understand how STN-DBS alleviates seizures in motor epilepsy mice, we implanted ipsilateral electrodes into the STN and simultaneously employed chemogenetic AAV with CNO to selectively activate the STN-SNr projections (Figures 6A,B). Histological data confirmed the DBS location and the mCherry-expressing neurons in the STN and SNr (Figure 6C). The representative EEG and the corresponding power spectrum are shown in Figure 6D. The EEG analysis demonstrated that, compared to sham-DBS, STN-DBS significantly decreased the spike frequency, amplitude, and power spectral density of motor seizures. In addition, similar to STN-DBS, STN-DBS + mCherry served as a vehicle control and showed a decrease in seizure activities, while mice in the STN-DBS + hM3Dq group showed almost no rescue effects and had significantly higher spike frequency (Figure 6E), amplitude (Figure 6F), and EEG density (Figure 6G) compared to those in the STN-DBS and STN-DBS + mCherry groups. In addition, STN-DBS significantly prolonged the latency to GSs, reduced the numbers of FSs and GSs, and delayed the development of behavioral seizure stages, with STN-DBS + mCherry exhibiting similar results. Conversely, mice in the STN-DBS + hM3Dq group had distinctly short latency to FSs (Figure 6H) and GSs (Figure 6I), higher numbers of FSs (Figure 6J) and GSs (Figure 6K; Supplementary Figure S3B), and more rapid development of seizure stages (Figure 6L) compared to those in the STN-DBS and STN-DBS + mCherry groups, except that there was no significant difference in the number of FSs between the STN-DBS + hM3Dq and STN-DBS + mCherry groups. Overall, we found that activation of the STN-SNr circuit can eliminate the benefits of high-frequency STN-DBS, suggesting that STN-DBS may improve seizure activity by inhibiting the STN-SNr circuit.

### Targeting SNr orexin receptors attenuates seizure activities

**Figure 7:**
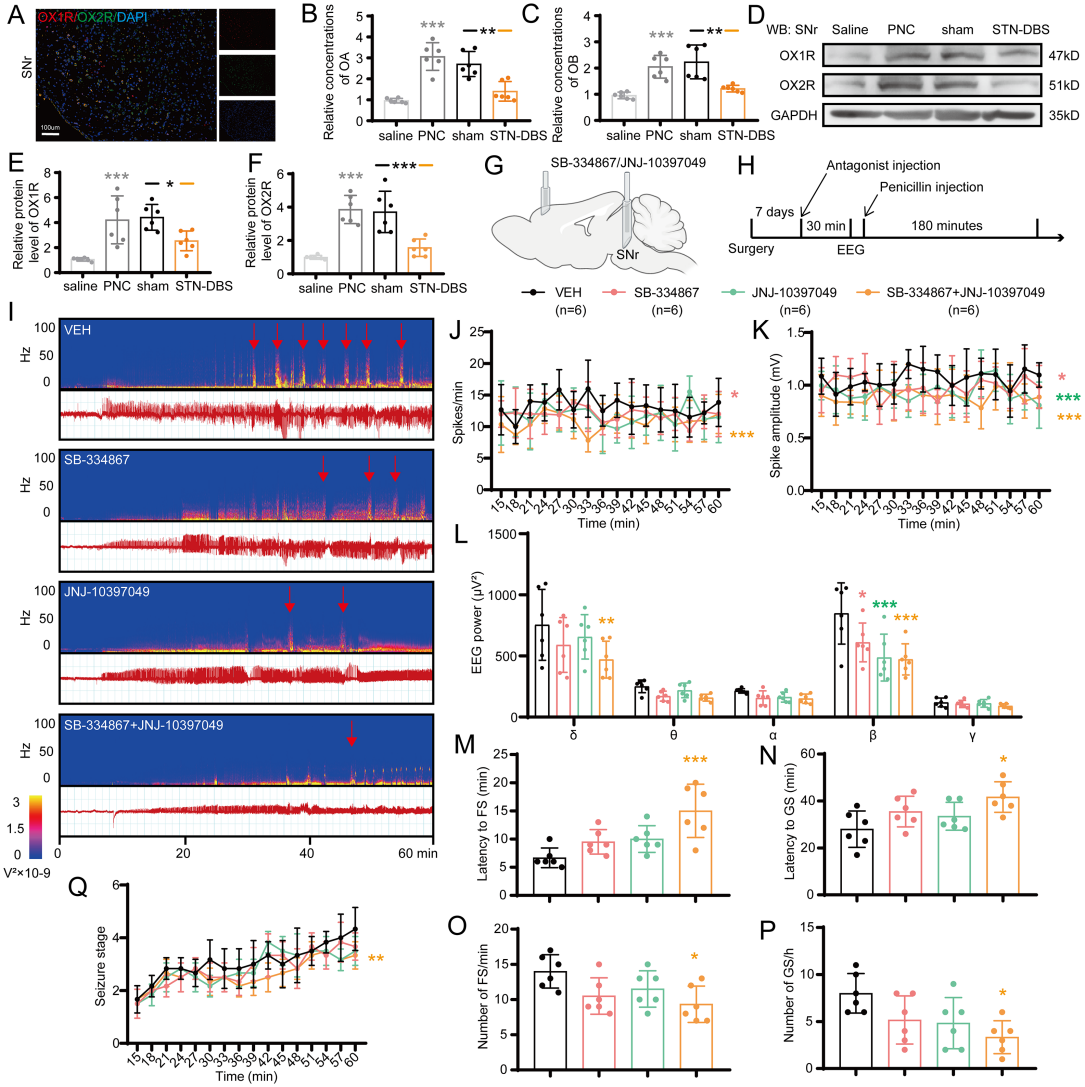
Modulating orexin receptors type 1 (OX1R) and 2 (OX2R) in substantia nigra pars reticulata (SNr) alleviates seizures. **(A)** Immunofluorescence analysis was performed using antibodies against OX1R (red) and OX2R (green) in brain sections of the SNr. Nuclei were fluorescently labeled with DAPI (blue). Representative image confirms the expression and co-localization of OX1R and OX2R in the SNr. Scale bar = 100 μm. Relative concentrations of **(B)** orexin A (OA) and **(C)** orexin B (OB) in the SNr, as detected by ELISA. Western blot analysis with **(D)** representative image and quantification of **(E)** OX1R and **(F)** OX2R levels in the SNr. *p < 0.05, **p < 0.01, ***p < 0.001. Gray asterisk indicates comparison of penicillin (PNC) and saline groups; black asterisk with black and orange horizontal lines to the left and right indicates comparison of the PNC + sham STN-DBS and PNC + real STN-DBS (ipsilateral, 130 Hz) groups. **(G,H)** Scheme and time course of the experiment for the efficacy of orexin receptor antagonists in motor epileptic mice. **(I)** Representative EEG spectra power and raw EEG; red arrowheads indicate generalized seizure (GS) onset. Effects of orexin receptor antagonist injection in the SNr on **(J)** spike frequency, **(K)** spike amplitude, **(L)** spectral power density, **(M)** latency to FS, **(N)** latency to GS, **(O)** number of focal seizure (FS), **(P)** number of GS, and **(Q)** development of seizure stage. *p < 0.05, **p < 0.01, ***p < 0.001. Colored asterisk indicates comparison of the corresponding group and the vehicle group. Data are presented as means ± SD. Detailed statistical methods and data are provided in Supplementary materials.

Since STN-DBS and optogenetic/chemogenetic inhibition of STN-SNr projections were sufficient for seizure rescue in motor epileptic mice, we examined whether a similar effect might be achieved using a molecular target. Orexin and its receptors are expressed in SNr (Korotkova et al., 2002; Li et al., 2019), and several studies found their important role in epilepsy (Zhu et al., 2015; Roundtree et al., 2016; Kordi Jaz et al., 2017). First, we reconfirmed the expression and co-localization of OX1R and OX2R in the SNr (Figure 7A). Next, to further explore the expression change of orexin and its receptors in the SNr following penicillin injection and STN-DBS, ELISA was used to detect the expression of OA and OB, considering their small molecular weight of 15kD. Western blot (WB) was used to evaluate the expressions of OX1R and OX2R. The results showed that the concentrations of OA and OB in the SNr were significantly increased after penicillin injection. STN-DBS reversed the enhancement of OA and OB in motor epilepsy mice, whereas sham-DBS had no effect (Figures 7B,C). Representative WB plot is shown in Figure 7D. Meanwhile, quantitative analysis found a similar expression change of OX1R and OX2R (Figures 7E,F). These data indicated that the expressions of orexin and its receptors are enhanced during seizures, and STN-DBS can reduce the expression of orexin and its receptors, which may cause reduced binding of orexin and its receptors. Next, we aimed to test whether the OX1R antagonist, SB-334867, and the OX2R antagonist, JNJ-10397049, could attenuate seizure activities in the motor epilepsy model (Figures 7G,H). A typical EEG and the corresponding power spectrum are shown in Figure 7I. EEG analysis demonstrated that selective inhibition of OX1R and dual inhibition of orexin receptors significantly decreased the spike frequency (Figure 7J), amplitude (Figure 7K), and EEG power (Figure 7L), while inhibition of OX2R failed to reduce spike frequency but lowered the spike amplitude and EEG power. In addition, for the behaviors, only dual inhibition substantially prolonged the latency to FSs (Figure 7M) and GSs (Figure 7N), decreased the numbers of FSs (Figure 7O) and GSs (Figure 7P; Supplementary Figure S3C), and delayed the seizure progression (Figure 7Q), while there was no statistically significant difference between OX1R antagonist or OX2R antagonist and vehicle. Together, these data indicate that targeting orexin receptors in SNr circuits may offer a potential therapeutic approach to alleviate seizure activities in motor epilepsy.

## Discussion

**Figure 8:**
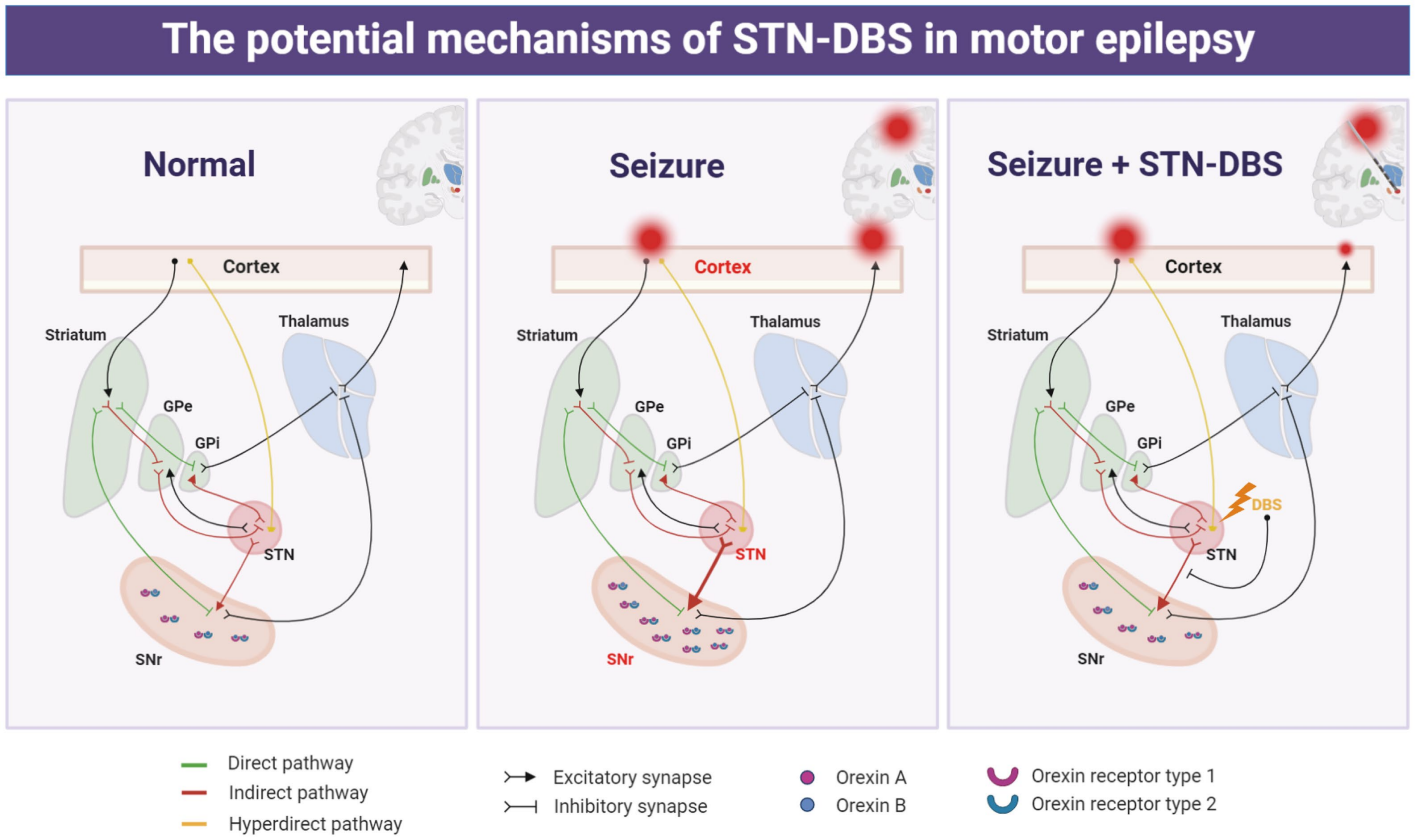
Schematic representation of the potential mechanism of action of deep brain stimulation of the subthalamic nucleus (STN-DBS) in motor epilepsy.

Seizures that emerge from the motor cortex networks are associated with considerable impairment. Regrettably, the implicated brain areas and causative processes underpinning the neural circuits have not been adequately studied. We found that STN, a specific site within the interconnected cortico-subcortical network in sensory-motor integration and motor control, is involved in the propagation network of focal motor seizures. Furthermore, high-frequency electrical stimulation of the STN can profoundly alleviate the motor cortex epileptic activity, implying that the STN may be a preferable target of DBS for motor seizures. Additionally, by utilizing optogenetics and chemogenetics, we showed that selectively suppressing the excitatory neurons in the STN may be advantageous for seizure management, and the STN-SNr circuit-specific mechanism contributes to the causal underpinnings of motor seizures. This suggests that STN-SNr circuits play a crucial role in reducing seizures in motor epilepsy and that STN-DBS may lower motor seizure activity by inhibition of the STN-SNr projections (Figure 8). Finally, we discovered molecular targets capable of influencing the STN-SNr circuits and showed that orexin receptor antagonists that target SNr neurons rescue seizure activity (Figure 8). Taken together, we not only discovered the circuit pathways responsible for seizure activity in motor epileptic mice but also proposed that employing STN-DBS or orexin receptor antagonists capable of modulating STN-SNr circuits offers an attractive treatment for epilepsy originating from the motor cortex.

STN is a well-known clinical target for therapeutic neurostimulation in movement disorders, including Parkinson’s disease and dystonia (Benabid et al., 2001; Weaver et al., 2009; Ryvlin and Jehi, 2022; Xue et al., 2022). Because of its surgical accessibility, this structure might be a viable option for targeted epilepsy treatment. Although the significance of the STN in epilepsy has received less attention, there is still evidence for it. On the one hand, some small-scale pilot clinical investigations have found it to be effective in the treatment of seizures, particularly focal motor seizures. Benabid et al. initially reported a case of a 5-year-old girl with drug-resistant epilepsy who underwent unilateral high-frequency STN-DBS, which was associated with an 80% reduction in seizure frequency as well as improvement in motor and cognitive function over a 2.5-year follow-up (Benabid et al., 2002). Other minor, uncontrolled investigations have also revealed a > 50% decrease of seizures (Chabardes et al., 2002; Handforth et al., 2006; Lee et al., 2006). Ren et al. investigated the modulatory effects of STN-DBS at various stimulation frequencies in seven patients with refractory focal motor seizures. In particular, low-frequency (20 Hz) or high-frequency (100/130 Hz) stimulation increased or decreased epileptic activity produced from motor regions (Ren et al., 2020). On the other hand, some experimental studies based on rodent epileptic models also found that STN is a promising therapeutic target of electrical stimulation for suppressing seizures. Vercueil et al. observed that 130-Hz, 60-μs STN-DBS suppressed seizures in a rat model of genetic absence epilepsy but only with bilateral stimulation (Vercueil et al., 1998). In addition, Lado et al. demonstrated that 130-Hz stimulation of the STN increased seizure threshold in a flurothyl-induced epileptic model, while a higher frequency of 260 or 800 Hz had no effect or even lowered the threshold (Lado et al., 2003). Additionally, STN-DBS was found to be effective in blocking seizures in some other models, such as those developed using kainic acid injection (Usui et al., 2005) or amygdaloid kindling (Shi et al., 2006). In summary, consistent with previous studies, our study also found that high-frequency stimulation was effective for motor epilepsy, while low-and medium-frequency stimulation did not improve or alleviate seizure control. High-frequency stimulation parameters were not studied because most studies also focused on high-frequency stimulation at around 130 Hz. There is also some controversy regarding the effects of unilateral and bilateral stimulation. There were many inconsistencies in previous studies, with some reporting that both unilateral and bilateral stimulation are effective and others suggesting that only bilateral stimulation is effective. Our study found that unilateral (ipsilateral) STN-DBS could effectively reduce epileptic activity, and bilateral stimulation showed similar or even better efficacy, but there was no statistically significant advantage compared to unilateral STN-DBS. Accordingly, the current and previous studies have consistently proven the effectiveness of STN-DBS for the treatment of motor epilepsy. Although previous studies have only identified this phenomenon, the underlying mechanism of the effects of STN-DBS on epilepsy remains unclear. This was the focus of our study. This study is the first to illustrate the circuit and molecular mechanism of STN-DBS for the treatment of motor epilepsy.

With regard to the circuit, STN-SNr projections are part of the classical basal ganglia pathways. SNr is composed of approximately 90% GABAergic neurons and receives a monosynaptic glutamatergic input from the STN (Shen and Johnson, 2006; Deniau et al., 2007). SNr is a well-established nigral inhibitory system, and it has been identified to have a seizure gating function (Gale, 1988). There is adequate evidence that direct inhibition of the SNr, *via* GABAergic drugs, GABAergic cell transplant, electrical stimulation, or optogenetic silencing or lesioning, can suppress various seizure types in animals (Garant and Gale, 1983; Thompson and Suchomelova, 2004; Shi et al., 2006; Castillo et al., 2008; Tollner et al., 2011; Gey et al., 2016; Wicker et al., 2019). Meanwhile, some previous studies have found that inhibition of the STN and its monosynaptic glutamatergic input to SNr can attenuate seizure susceptibility (Dybdal and Gale, 2000; Usui et al., 2005; Backofen-Wehrhahn et al., 2018). In line with previous research, our study demonstrated that photo-inhibition of STN or photo−/chemo-inhibition of the STN-SNr circuit can satisfactorily attenuate motor FSs and secondary GSs in models with epilepsy arising from M1. Moreover, we found that when high-frequency STN-DBS plus chemogenetic activation of the STN-SNr pathway was used, the original improvement of electrical stimulation was canceled out, suggesting that STN-DBS may ameliorate seizures by regulating the excitability of the STN-SNr loop. This study revealed the partly circuit-related mechanism of improvement by STN-DBS in motor epilepsy. However, considering that this study only focused on STN-SNr projections, it is not known whether other projections of the STN are also involved in the improvement of epileptic activity by STN-DBS. In addition, it is necessary to further explore the downstream nucleus that is regulated by SNr and how it ultimately affects epilepsy.

In addition, orexin, another focus of this study, is secreted by a group of neurons in the hypothalamus that are located closest to the STN and project to the STN and SNr (Peyron et al., 1998). Several studies have found that the orexin pathway plays a vital role in various seizures (Samzadeh et al., 2020; Celli and Luijtelaar, 2022; Konduru et al., 2022). Previous studies have observed the expression and co-localization of OX1R and OX2R in the STN and SNr (Korotkova et al., 2002; Li et al., 2019). Therefore, we were naturally aware of whether the decrease of seizure activity by STN-DBS in motor epilepsy is related to the regulation of the orexin pathway. Our results show that STN-DBS can restore (downregulate) the elevated levels of OA, OB, OX1R, and OX2R associated with seizures in the SNr. Furthermore, antagonizing both OX1R and OX2R simultaneously can play a similar role as high-frequency STN-DBS and attenuate motor epilepsy. We identified the molecular targets that modulate the seizure activity in the STN-SNr circuit, which has the potential to be a drug target for the treatment of motor epilepsy.

Overall, our results demonstrate that high-frequency STN-DBS attenuates motor seizures *via* inhibition of the STN-SNr circuits. The orexin system plays a vital role during electrical stimulation, and orexin receptor antagonists have a therapeutic potential in suppressing motor seizure activities. This study contributes to a better understanding of the current network theory of epilepsy and the mechanism of STN-DBS.

## Data availability statement

The original contributions presented in the study are included in the article/Supplementary material, further inquiries can be directed to the corresponding authors.

## Ethics statement

The animal study was reviewed and approved by the Animal Advisory Committee of the Beijing Tiantan Hospital.

## Author contributions

TX: conceptualization, investigation, methodology, and wrote the original draft. SW: investigation, methodology, and wrote the revised draft. SC: data curation and investigation. HW: formal analysis and software. CL: investigation and validation. LS: visualization. YB: data curation. CZ: methodology and data curation. CH: conceptualization, funding acquisition, wrote the original draft, and supervision. JZ: conceptualization, funding acquisition, project administration, and supervision. All authors contributed to the article and approved the submitted version.

## Funding

This work was supported by the National Natural Science Foundation of China [grant numbers 82171442 and 81901314].

## Conflict of interest

The authors declare that the research was conducted in the absence of any commercial or financial relationships that could be construed as a potential conflict of interest.

## Publisher’s note

All claims expressed in this article are solely those of the authors and do not necessarily represent those of their affiliated organizations, or those of the publisher, the editors and the reviewers. Any product that may be evaluated in this article, or claim that may be made by its manufacturer, is not guaranteed or endorsed by the publisher.

## Abbreviations

AAV: adeno-associated virus
ANT: anterior nucleus of the thalamus
DBS: deep brain stimulation
FS: focal seizure
GS: generalized seizure
M1: primary motor cortex
OA: Orexin A
OB: Orexin B
OX1R: orexin receptor type 1
OX2R: orexin receptor type 2
PBS: phosphate buffer solution
PFA: paraformaldehyde
STN: subthalamic nucleus
SNR: substantia nigra pars reticulata
