# Supplementary material for: Subthalamic nucleus stimulation attenuates motor seizures via modulating the nigral orexin pathway
Source: Front Neurosci. 2023 May 5;17:1157060. doi: 10.3389/fnins.2023.1157060 (PMC10196042; doi:10.3389/fnins.2023.1157060)
Supplement: Supplementary file 1 [file Data_Sheet_1.PDF]

Subthalamic Nucleus Stimulation Attenuates Motor Seizures via Modulating the Nigral Orexin Pathway

Supplementary materials

Supplemental figure 1. Verification of motor epileptic model and STN-SNr circuit. (A) Representative raw EEG in motor epileptic mouse with (a) baseline, (b) preictal, (c) focal seizure, (d) secondary generalized seizure and (e) suppression state after generalized seizure (green rectangle). **(B)** Fiber photometry of neural dynamics during motor seizures after triple injection of AAVs in M1, STN and SNr, confirming the activation of these brain regions during motor seizures. rAAV-CaMKIIa-GCaMp6m was injected into M1, STN, and rAAV-hSyn-GCaMp6m was used for SNr in wild-type C57/BL6 mice. **(C)** Representative images of the STN electrode location. Scale bar = 500 μm. **(D)** Adjusted number of GS/h for figure 1N. The time was corrected according to latency to GS, and to be specific, adjusted number of GS/h = total number of GS / (3h – latency to GS). **$p < 0.01$. Data are presented as means ± SD. Colored asterisk indicates the comparison of the corresponding group and the penicillin + sham STN-DBS group. Detailed statistical methods and data are provided in Supplementary table 3. **(E)** Immunofluorescence analysis was performed with antibodies against c-Fos (red) and NeuN (green) in brain sections of M1, STN and SNr. Nuclei were fluorescently labeled with DAPI (blue), scale bar = 500 μm for whole brain perspective; scale bar = 50 μm for zoomed perspective. Verification of the classic STN-SNr projections by **(F)** anterograde and **(G)** retrograde tracer, scale bar = 100 μm.

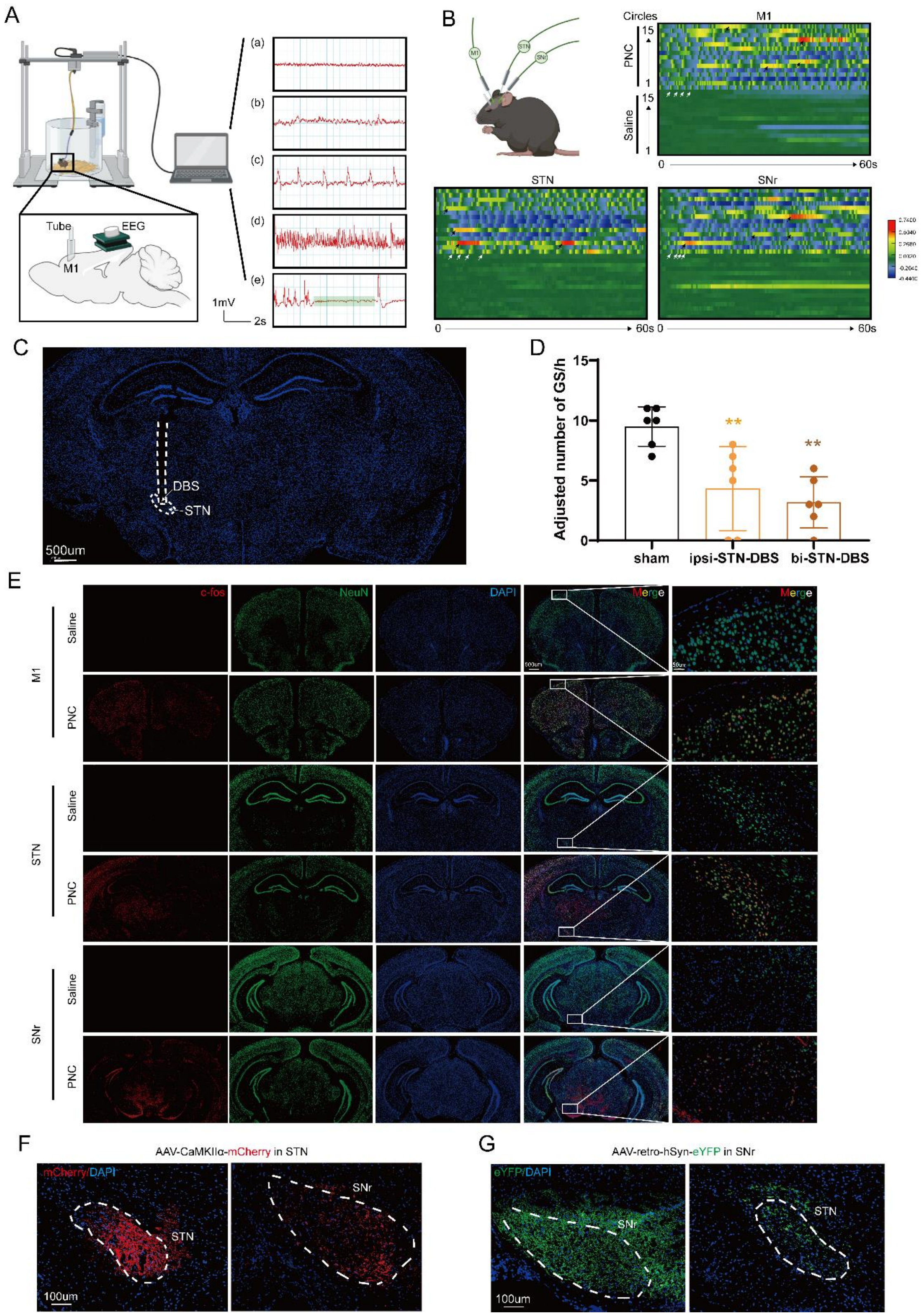

A
(a)
(b)
(c)
(d)
(e)
Tube
EEG
M1
1mV
2s
B
Circles
M1
PNC
Saline
15
1
0
60s
STN
SNr
C
DBS
-STN
500um
D
Adjusted number of GS/h
sham
ipsi-STN-DBS
bi-STN-DBS
E
c-fos
NeuN
DAPI
Merge
Saline
PNC
F
AAV-CaMKIIα-mCherry in STN
mCherry/DAPI
100um
G
AAV-retro-hSyn-eYFP in SNr
eYFP/DAPI

Supplemental figure 2. Effects of chemogenetic regulation of the subthalamic nucleus-substantia nigra pars reticulata (STN-SNr) circuit at pre, CNO and post. (A-D) Effects of CNO treatment on the number of FS, latency to FS, number of GS and latency of GS. **(E-H)** Effects of chemogenetic activation on the number of FS, latency to FS, number of GS and latency of GS. **(I-L)** Effects of chemogenetic inhibition treatment on the number of FS, latency to FS, number of GS and latency of GS. */#P < 0.05, **/##P < 0.01, asterisk stands for comparison with pre; pound stands for comparison with post. Data are presented as means ± SD. Detailed statistical methods and data are provided in Supplementary materials.

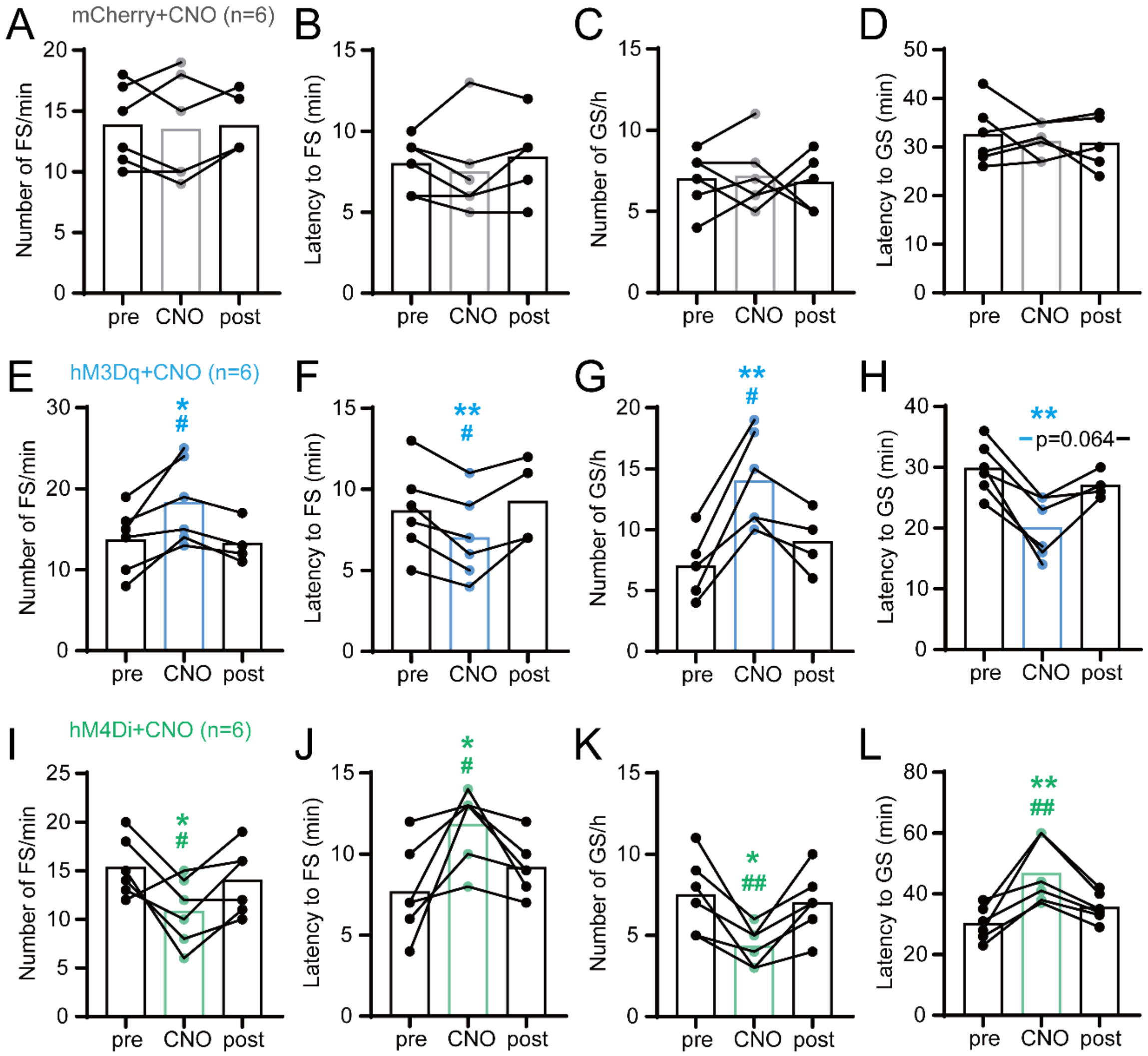

Supplemental figure 3. Adjusted number of GS/h for **(A)** figure 4K, **(B)** 6K and **(C)**7P. The time was corrected according to latency to GS, and to be specific, adjusted number of GS/h = total number of GS / (3h – latency to GS). *p < 0.05, **p < 0.01, ***p < 0.001. Data are presented as means ± SD. Colored asterisk indicates the comparison of the corresponding group and the control (first) group. Black asterisk with two different colored horizontal lines to the left and right represents comparison of the corresponding two groups. Detailed statistical methods and data are provided in Supplementary table 3.

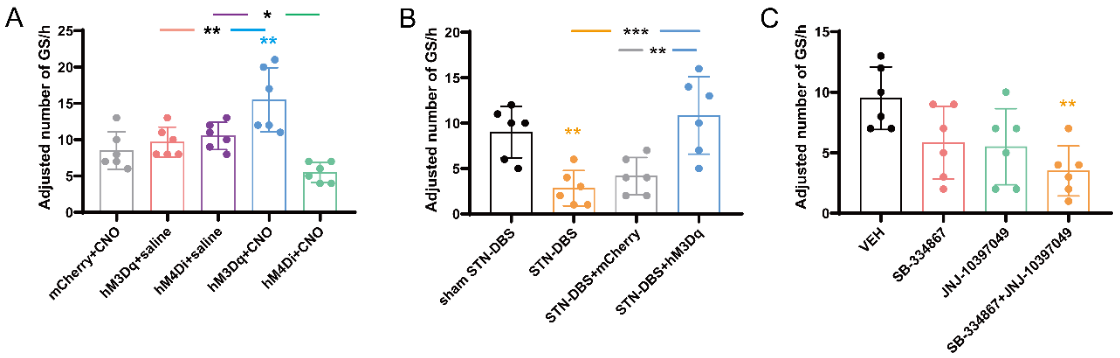

Supplemental table 1. Mice mortality and exclusion

Experiment	Group	Subtotal	Mortality (%)	Excluded	Included
Validation of model (EEG and IF)	Saline	3	0 (0)	0	3
	PNC	4	1 (25)	0	3
Validation of model (Ca^{2+} signal)	PNC	4	0 (0)	1 (inaccurate placement of fiber)	3
Anterograde trace	-	3	0 (0)	0	3
Retrograde trace	-	3	0 (0)	0	3
STN-DBS (Ca^{2+} signal)	Saline/ PNC	7	2 (29)	0	5
STN-DBS (EEG and IF)	PNC + sham STN-DBS	9	2 (22)	1 (no seizure)	6
	PNC + ipsilateral STN-DBS	7	1 (14)	0	6
	PNC + bilateral STN-DBS	9	1 (11)	2 (inaccurate placement of DBS)	6
STN ChR2	Blue light ON/OFF	10	3 (30)	2 (inaccurate placement of fiber)	5
	Yellow light ON/OFF	7	1 (14)	0	6
STN eNpHR	Yellow light ON/OFF	6	0 (0)	0	6
	Blue light ON/OFF	8	2 (25)	0	6
STN-SNr hM3Dq/ hM4Di	mCherry + CNO (pre/CNO/post)	11	0/1/2 (27)	2 (one for no seizure at CNO; one for poor condition after pre)	6
	hM3Dq + saline (pre/saline/post)	10	1/1/1 (30)	1 (cement shedding)	6
	hM4Di + saline (pre/saline/post)	9	0/1/1 (23)	1 (insufficient viral infection)	6
	hM3Dq + CNO (pre/CNO/post)	12	2/2/1 (42)	1 (plugged tube before CNO)	6
	hM4Di + CNO (pre/CNO/post)	10	1/0/1 (20)	1 (one for maybe infection before pre after surgery; one for insufficient viral infection)	6
STN-SNr ChR2/eYFP	ChR2 + blue light ON/OFF	8	2 (25)	0	6
	eYFP + blue light ON/OFF	7	0 (0)	1 (losing too much weight)	6
STN-SNr eNpHR/eYFP	eNpHR + yellow light ON/OFF	7	1 (14)	0	6
	eYFP + yellow light ON/OFF	8	2 (25)	0	6
STN-DBS hM3Dq	Sham STN-DBS [#]	8	2 (25)	0	6
	STN-DBS [*]	8	1 (12.5)	1 (inaccurate placement of DBS)	6
	STN-DBS + mCherry	7	1 (14)	0	6
	STN-DBS + hM3Dq	10	2 (20)	2 (poor condition;	6

				insufficient viral infection)	
Orexin receptor 1/2 (IF)	-	3	0 (0)	0	3
Orexin A/B and orexin receptor 1/2 (ELISA, WB)	Saline	6	0 (0)	0	6
	PNC	7	1 (14)	0	6
	Sham STN-DBS	Use above-mentioned mice [#]			
	STN-DBS	Use above-mentioned mice [*]			
Antagonists	Vehicle	7	1 (14)	0	6
	SB-334867	7	1 (14)	0	6
	JNJ-10397049	6	0 (0)	0	6
	SB-334867 + JNJ-10397049	7	0 (0)	1 (no seizure)	6
**Total**		**238**	**42 (18)**	**18**	**178**

Supplemental table 2. Resource Identifiers for antibodies

Target antigen	c-fos	Orexin A	Orexin B	Orexin receptor 1	Orexin receptor 2
Antibody name	Anti-c-Fos antibody	Anti-Orexin A antibody	Anti-Orexin B antibody	Orexin Receptor 1 (HCRTR1) Rabbit Polyclonal Antibody	RABBIT ANTI-OREXIN-2 RECEPTOR AFFINITY PURIFIED POLYCLONAL ANTIBODY
Reactivity	Human, Porcine, Rat, Cow, Mouse, Horse	Mouse, Rat	Mouse, Rat	Rat, Mouse, Human	Rat, Mouse, Human
Host	Mouse	Rabbit	Rabbit	Rabbit	Rabbit
Vendor	Abcam	Abcam	Abcam	OriGene	Millipore
Cat number	ab208942	ab255294	ab255293	TA376968	AB3094
RRID	AB_2747772	N/A	N/A	N/A	AB_91358
Proper Citation	Abcam Cat# ab208942, RRID: AB_2747772	Abcam Cat# ab255294	Abcam Cat# ab255293	OriGene Cat# TA376968	Millipore Cat# AB3094, RRID: AB_91358
Reference	Reference (45)	Reference (0)	Reference (0)	Reference (2)	Reference (3)
Clonality	Monoclonal	Monoclonal	Monoclonal	Polyclonal	Polyclonal
Clone ID	2H2	EPR22803-259	EPR22803-18	N/A	N/A
Comments	WB, ICC/IF, IHC	Dot blot, IHC-P, IHC-Fr, ELISA	Dot blot, IHC-P, IHC-Fr, ELISA	IF, WB	IF, ELISA, WB

Applicate dilution	IF=1:1000	ELISA= 1:1000	ELISA= 1:1000	IF=1:100<br>WB=1:1000	IF=1:200<br>WB=1:500

Supplemental table 3. Statistical table

	Description	Normality tests	Test used	Stat-value	One- or two-tailed P value?
Fig. 1H	Spikes/min	Passed	Two-way repeated ANOVA with Geisser-Greenhouse's correction followed by post hoc Tukey's tests	Interaction: F (30, 225) = 2.681, P<0.0001;<br>Time: F (6.847, 102.7) = 11.63, P<0.0001;<br>Group: F (2, 15) = 97.35, P<0.0001;<br>For sham vs. ipsi, P<0.0001;<br>For sham vs. bi, P<0.0001.	Two-tailed
Fig. 1I	Spike amplitude (mV)	Passed	Two-way repeated ANOVA with Geisser-Greenhouse's correction followed by post hoc Tukey's tests	Interaction: F (30, 225) = 1.519, P=0.0477;<br>Time: F (7.071, 106.1) = 1.560, P=0.1547;<br>Group: F (2, 15) = 7.254, P=0.0063;<br>For sham vs. ipsi, P= 0.0006;<br>For sham vs. bi, P= 0.0123.	Two-tailed
Fig. 1J	EEG power (uV^2)	Passed	Two-way ANOVA followed by post hoc Tukey's tests	Interaction: F (8, 75) = 6.847, P<0.0001;<br>Band: F (4, 75) = 238.5, P<0.0001;<br>Group: F (2, 75) = 35.30, P<0.0001;<br>For δ, sham vs. ipsi, P<0.0001;<br>For δ, sham vs. bi, P<0.0001;<br>For β, sham vs. ipsi, P<0.0001;<br>For β, sham vs. bi, P<0.0001;<br>For β, ipsi vs. bi, P= 0.0002;<br>For γ, sham vs. bi, P<0.0443.	Two-tailed
Fig. 1K	Latency to FS (min)	Not necessary	Kruskal-Wallis test followed by post hoc Dunn's tests	KW statistic=7.997, P=0.0122;<br>For sham vs. ipsi, P= 0.0502;<br>For sham vs. bi, P= 0.0371.	Two-tailed
Fig. 1L	Latency to GS (min)	Not necessary	Kruskal-Wallis test followed by post hoc Dunn's tests	KW statistic=8.772, P=0.0063;<br>For sham vs. ipsi, P= 0.0447;<br>For sham vs. bi, P= 0.0222.	Two-tailed
Fig. 1M	Number of FS/min	Not necessary	Kruskal-Wallis test followed by post hoc Dunn's tests	KW statistic=8.272, P=0.0095;<br>For sham vs. ipsi, P= 0.0466;<br>For sham vs. bi, P= 0.0318.	Two-tailed
Fig. 1N	Number of GS/h	Not necessary	Kruskal-Wallis test followed by post hoc Dunn's tests	KW statistic=11.50, P=0.0005;<br>For sham vs. ipsi, P= 0.0272;<br>For sham vs. bi, P= 0.0044.	Two-tailed
Fig. 1O	Seizure stage	Passed	Two-way repeated ANOVA with Geisser-Greenhouse's correction followed by post hoc Tukey's tests	Interaction: F (30, 225) = 1.545, P=0.0411;<br>Time: F (6.506, 97.59) = 19.84, P<0.0001;<br>Group: F (2, 15) = 3.432, P=0.0593;<br>For sham vs. ipsi, P= 0.1758;<br>For sham vs. bi, P= 0.1362.	Two-tailed
Fig. 1Q	Relative fluorescent intensity of c-fos	Passed	Two-way ANOVA followed by post hoc Tukey's tests	Interaction: F (4, 45) = 7.986, P<0.0001;<br>Brain region: F (2, 45) = 5.378, P=0.0080;<br>Group: F (2, 45) = 404.5, P<0.0001;<br>For M1, sham vs. ipsi, P<0.0001;<br>For M1, sham vs. bi, P<0.0001;	Two-tailed

				For STN, sham vs. ipsi, P<0.0001;<br>For STN, sham vs. bi, P<0.0001;<br>For STN, ipsi vs. bi, P= 0.0416;<br>For SNr, sham vs. ipsi, P<0.0001;<br>For SNr, sham vs. bi, P<0.0001.	
Fig. 2E	Spikes/min	Passed	Two-way repeated ANOVA with Geisser-Greenhouse's correction followed by post hoc Sidak tests	Interaction: F (15, 135) = 3.616, P<0.0001;<br>Time: F (4.689, 42.20) = 4.071, P=0.0049;<br>Group: F (1, 9) = 23.98 , P=0.0009;<br>For 24 min, blue vs. yellow, P= 0.0342;<br>For 54 min, blue vs. yellow, P= 0.0158.	Two-tailed
Fig. 2F	Spike amplitude (mV)	Passed	Two-way repeated ANOVA with Geisser-Greenhouse's correction followed by post hoc Sidak tests	Interaction: F (15, 135) = 5.568, P<0.0001;<br>Time: F (5.236, 47.13) = 4.921, P=0.0009;<br>Group: F (1, 9) = 9.365, P=0.0136;<br>For 24 min, blue vs. yellow, P= 0.0122.	Two-tailed
Fig. 2G	EEG power (uV^2)	Passed	Two-way ANOVA followed by post hoc Tukey's tests	Interaction: F (8, 95) = 4.918, P<0.0001;<br>Band: F (4, 95) = 74.21, P<0.0001;<br>Group: F (2, 95) = 25.50, P<0.0001;<br>For δ, blue vs. no, P<0.0001;<br>For δ, blue vs. yellow, P<0.0001;<br>For θ, blue vs. no, P=0.0052;<br>For β, blue vs. no, P=0.0001;<br>For β, blue vs. yellow, P= 0.0020.	Two-tailed
Fig. 2H	Number of FS/min	Passed	Paired and Unpaired T tests	For blue vs. no, paired t=3.359, df=4, P=0.0283;<br>For blue vs. yellow, unpaired t=4.504, df=9, P=0.0015.	Two-tailed
Fig. 2I	Number of GS/h	Passed	Paired and Unpaired T tests	For blue vs. no, paired t=2.922, df=4, P=0.0432;<br>For blue vs. yellow, unpaired t=3.061, df=9, P=0.0135.	Two-tailed
Fig. 2J	Seizure stage	Unpassed	Mann-Whitney U test	Mann-Whitney U= 49, P=0.0021.	Two-tailed
			Pilot two-way repeated ANOVA with Geisser-Greenhouse's correction followed by post hoc Sidak tests	Interaction: F (15, 135) = 2.414 P=0.0039<br>Time: F (4.877, 43.89) = 21.09 P<0.0001<br>Group: F (1, 9) = 63.91 P<0.0001<br>For 48 min, blue vs. yellow, P= 0.0264;<br>For 60 min, blue vs. yellow, P= 0.0083	
Fig. 3E	Spikes/min	Passed	Two-way repeated ANOVA with Geisser-Greenhouse's correction followed by post hoc Sidak tests	Interaction: F (15, 150) = 2.363, P=0.0045;<br>Time: F (15, 150) = 1.716, P=0.0005;<br>Group: F (1, 10) = 30.34, P=0.0003;<br>For 30 min, blue vs. yellow, P= 0.0295;<br>For 42 min, blue vs. yellow, P= 0.0451;<br>For 54 min, blue vs. yellow, P= 0.0159.	Two-tailed
Fig. 3F	Spike amplitude (mV)	Passed	Two-way repeated ANOVA with Geisser-Greenhouse's correction	Interaction: F (15, 150) = 2.507, P=0.0025;<br>Time: F (4.679, 46.79) = 7.213, P<0.0001;<br>Group: F (1, 10) = 8.011, P=0.0178;	Two-tailed

			followed by post hoc Sidak tests	For 24 min, blue vs. yellow, P= 0.0464.	
Fig. 3G	EEG power (uV^2)	Passed	Two-way ANOVA followed by post hoc Tukey’s tests	Interaction: F (8, 105) = 1.660, P=0.1168;<br>Band: F (4, 105) = 77.15, P<0.0001;<br>Group: F (2, 105) = 17.95, P<0.0001;<br>For δ, yellow vs. no, P=0.0039;<br>For δ, yellow vs. blue, P=0.0102;<br>For α, yellow vs. blue, P=0.0401;<br>For β, yellow vs. no, P=0.0001;<br>For β, yellow vs. blue, P<0.0001;<br>For γ, yellow vs. no, P=0.0478.	Two-tailed
Fig. 3H	Number of FS/min	Passed	Paired and Unpaired T tests	For yellow vs. no, paired t=3.997, df=5, P=0.0104;<br>For yellow vs. blue, unpaired t=3.654, df=10, P=0.0044.	Two-tailed
Fig. 3I	Number of GS/h	Passed	Paired and Unpaired T tests	For yellow vs. no, paired t=3.782, df=5, P=0.0129;<br>For yellow vs. blue, unpaired t=1.980, df=10, P=0.0759.	Two-tailed
Fig. 3J	Seizure stage	Unpassed	Mann-Whitney U test	Mann-Whitney U= 46.50, P=0.0013.	Two-tailed
			Pilot two-way repeated ANOVA with Geisser-Greenhouse's correction followed by post hoc Sidak tests	Interaction: F (15, 150) = 0.9394, P=0.5223;<br>Time: F (5.877, 58.77) = 14.29, P<0.0001;<br>Group: F (1, 10) = 34.39, P=0.0002.	
Fig. 4E	Spikes/min	Passed	Two-way repeated ANOVA with Geisser-Greenhouse's correction followed by post hoc Sidak tests	Interaction: F (60, 375) = 1.504, P=0.0131;<br>Time: F (8.628, 215.7) = 0.8088, P=0.6039;<br>Group: F (4, 25) = 19.27, P<0.0001;<br>For mCherry+CNO vs. hM3Dq+CNO, P<0.0001;<br>For mCherry+CNO vs. hM4Di+CNO, P<0.0001;<br>For hM3Dq+saline vs. hM3Dq+CNO, P<0.0001;<br>For hM4Di+saline vs. hM4Di+CNO, P<0.0001.	Two-tailed
Fig. 4F	Spike amplitude (mV)	Passed	Two-way repeated ANOVA with Geisser-Greenhouse's correction followed by post hoc Sidak tests	Interaction: F (60, 375) = 1.11, P=0.2767;<br>Time: F (8.333, 208.3) = 5.087, P<0.0001;<br>Group: F (4, 25) = 181.8, P<0.0001;<br>For mCherry+CNO vs. hM3Dq+CNO, P<0.0001;<br>For mCherry+CNO vs. hM4Di+CNO, P<0.0001;<br>For hM3Dq+saline vs. hM3Dq+CNO, P<0.0001;<br>For hM4Di+saline vs. hM4Di+CNO, P<0.0001.	Two-tailed
Fig. 4G	EEG power (uV^2)	Passed	Two-way ANOVA followed by post hoc Tukey’s tests	Interaction: F (16, 125) = 5.660, P<0.0001;<br>Band: F (4, 125) = 203.3, P<0.0001;<br>Group: F (4, 125) = 29.53, P<0.0001;<br>For δ, mCherry+CNO vs. hM3Dq+CNO, P<0.0001;<br>For δ, hM3Dq+saline vs. hM3Dq+CNO, P<0.0001;<br>For δ, hM4Di+saline vs. hM4Di+CNO, P=0.0067;	Two-tailed

				For θ, mCherry+CNO vs. hM3Dq+CNO, P=0.0195;<br>For β, mCherry+CNO vs. hM3Dq+CNO, P=0.0048;<br>For β, mCherry+CNO vs. hM4Di+CNO, P<0.0001;<br>For β, hM3Dq+saline vs. hM3Dq+CNO, P=0.0109;<br>For β, hM4Di+saline vs. hM4Di+CNO, P=0.0038.	
Fig. 4H	Latency to FS (min)	Passed	One-way ANOVA followed by post hoc Tukey's tests	F (4, 25) = 3.970, P=0.0125;<br>For mCherry+CNO vs. hM4Di+CNO, P=0.0338;<br>For hM4Di+saline vs. hM4Di+CNO, P=0.0338.	Two-tailed
Fig. 4I	Latency to GS (min)	Passed	One-way ANOVA followed by post hoc Tukey's tests	F (4, 25) = 14.35, P<0.0001;<br>For mCherry+CNO vs. hM3Dq+CNO, P=0.0384;<br>For mCherry+CNO vs. hM4Di+CNO, P=0.0023;<br>For hM4Di+saline vs. hM4Di+CNO, P=0.0004.	Two-tailed
Fig. 4J	Number of FS/min	Passed	One-way ANOVA followed by post hoc Tukey's tests	F (4, 25) = 11.62, P<0.0001;<br>For mCherry+CNO vs. hM3Dq+CNO, P=0.0123;<br>For mCherry+CNO vs. hM4Di+CNO, P=0.0290;<br>For hM4Di+saline vs. hM4Di+CNO, P=0.0381.	Two-tailed
Fig. 4K	Number of GS/h	Passed	One-way ANOVA followed by post hoc Tukey's tests	F (4, 25) = 13.59, P<0.0001;<br>For mCherry+CNO vs. hM3Dq+CNO, P=0.0003;<br>For hM3Dq+saline vs. hM3Dq+CNO, P=0.0018;<br>For hM4Di+saline vs. hM4Di+CNO, P=0.0358.	Two-tailed
Fig. 4L	Seizure stage	Passed	Two-way repeated ANOVA with Geisser-Greenhouse's correction followed by post hoc Sidak tests	Interaction: F (60, 375) = 1.089, 0.3139;<br>Time: F (8.234, 205.8) = 36.14, 0.0001;<br>Group: F (4, 25) = 58.35, 0.0001;<br>For mCherry+CNO vs. hM3Dq+CNO, P<0.0001;<br>For hM3Dq+saline vs. hM3Dq+CNO, P<0.0001.	Two-tailed
Fig. 5E	Spikes/min	Passed	Two-way repeated ANOVA with Geisser-Greenhouse's correction followed by post hoc Sidak tests	Interaction: F (15, 150) = 3.193, 0.0001;<br>Time: F (15, 150) = 4.769, 0.0020;<br>Group: F (1, 10) = 8.004, 0.0179;<br>For 30 min, ChR2 vs. eYFP, P= 0.0064.	Two-tailed
Fig. 5F	Spike amplitude (mV)	Passed	Two-way repeated ANOVA with Geisser-Greenhouse's correction followed by post hoc Sidak tests	Interaction: F (15, 150) = 1.844, P=0.0335;<br>Time: F (5.581, 55.81) = 1.994, 0.0866;<br>Group: F (1, 10) = 0.2267, P=0.6442.	Two-tailed
Fig. 5G	EEG power (uV^2)	Unpassed	Mann-Whitney U tests	For δ, Mann-Whitney U=5, P=0.0411;<br>For β, Mann-Whitney U=8, P=0.1320.	Two-tailed
Fig. 5H	Number of FS/min	Passed	Paired T test	t=4.183, df=5, P=0.0086.	Two-tailed
Fig. 5I	Number of GS/h	Passed	Paired T test	t=2.708, df=5, P=0.0424.	Two-tailed
Fig.	Seizure	Passed	Two-way repeated	Interaction: F (15, 150) = 2.003, P=0.0184;	Two-tailed

5J	stage		ANOVA with Geisser-Greenhouse's correction followed by post hoc Sidak tests	Time: F (5.503, 55.03) = 19.80, P<0.0001;<br>Group: F (1, 10) = 7.737, P=0.0194.	
Fig. 5K	Spikes/min	Passed	Two-way repeated ANOVA with Geisser-Greenhouse's correction followed by post hoc Sidak tests	Interaction: F (15, 150) = 1.742, P=0.0485;<br>Time: F (5.329, 53.29) = 4.083, P=0.0027;<br>Group: F (1, 10) = 0.5042, P=0.4939.	Two-tailed
Fig. 5L	Spike amplitude (mV)	Unpassed	Mann-Whitney U test	Mann-Whitney U=93, P=0.1930.	Two-tailed
			Pilot two-way repeated ANOVA with Geisser-Greenhouse's correction followed by post hoc Sidak tests	Interaction: F (15, 150) = 1.287, P=0.2169;<br>Time: F (5.614, 56.14) = 2.050, P=0.0781;<br>Group: F (1, 10) = 1.622, P=0.2317.	
Fig. 5M	EEG power (uV^2)	Passed	Two-way repeated ANOVA followed by post hoc Tukey's tests	Interaction: F (4, 50) = 2.802, P=0.0355;<br>Band: F (4, 50) = 39.48, P<0.0001;<br>Group: F (1, 50) = 6.645, P=0.0129;<br>For δ, P=0.0080;<br>For β, P=0.0628.	Two-tailed
Fig. 5N	Number of FS/min	Passed	Paired T test	t=3.371, df=5, P=0.0119.	Two-tailed
Fig. 5O	Number of GS/h	Passed	Paired T test	t=2.697, df=5, P=0.0429.	Two-tailed
Fig. 5P	Seizure stage	Passed	Two-way repeated ANOVA with Geisser-Greenhouse's correction followed by post hoc Sidak tests	Interaction: F (15, 150) = 1.842, P=0.0337;<br>Time: F (5.535, 55.35) = 20.89, P<0.0001;<br>Group: F (1, 10) = 7.327, P=0.0221.	Two-tailed
Fig. 6E	Spikes/min	Passed	Two-way repeated ANOVA with Geisser-Greenhouse's correction followed by post hoc Tukey's tests	Interaction: F (45, 300) = 0.7226, P=0.9069;<br>Time: F (8.530, 170.6) = 0.5626, P=0.8178;<br>Group: F (3, 20) = 24.11, P<0.0001;<br>For sham STN-DBS vs. STN-DBS, P<0.0001;<br>For STN-DBS vs. STN-DBS+hM3Dq, P<0.0001;<br>For STN-DBS+mCherry vs. STN-DBS+hM3Dq, P<0.0001.	Two-tailed
Fig. 6F	Spike amplitude (mV)	Passed	Two-way repeated ANOVA with Geisser-Greenhouse's correction followed by post hoc Tukey's tests	Interaction: F (45, 285) = 1.039, P=0.4119;<br>Time: F (7.867, 149.5) = 0.6779, P=0.7080;<br>Group: F (3, 19) = 9.480, P=0.0005;<br>For sham STN-DBS vs. STN-DBS, P=0.0030;<br>For STN-DBS vs. STN-DBS+hM3Dq, P=0.0014;<br>For STN-DBS+mCherry vs. STN-DBS+hM3Dq, P=0.0046.	Two-tailed
Fig.	EEG power	Passed	Two-way ANOVA	Interaction: F (12, 100) = 1.577, P=0.1104	Two-tailed

6G	(uV^2)		followed by post hoc Tukey's tests	Band: F (4, 100) = 98.13, P<0.0001<br>Group: F (3, 100) = 9.725, P<0.0001<br>For δ, sham STN-DBS vs. STN-DBS, P=0.0136;<br>For δ, STN-DBS vs. STN-DBS+hM3Dq, P=0.0006;<br>For δ, STN-DBS+mCherry vs. STN-DBS+hM3Dq, P=0.0056;<br>For β, sham STN-DBS vs. STN-DBS, P=0.0221;<br>For β, STN-DBS vs. STN-DBS+hM3Dq, P=0.0642;<br>For β, STN-DBS+mCherry vs. STN-DBS+hM3Dq, P=0.0028.	
Fig. 6H	Latency to FS (min)	Passed	One-way ANOVA followed by post hoc Tukey's tests	F (3, 20) = 5.335, P=0.0073;<br>For sham STN-DBS vs. STN-DBS, P=0.0628;<br>For STN-DBS vs. STN-DBS+hM3Dq, P=0.0204;<br>For STN-DBS+mCherry vs. STN-DBS+hM3Dq, P=0.0405.	Two-tailed
Fig. 6I	Latency to GS (min)	Passed	One-way ANOVA followed by post hoc Tukey's tests	F (3, 20) = 13.64, P<0.0001;<br>For sham STN-DBS vs. STN-DBS, P=0.0230;<br>For STN-DBS vs. STN-DBS+hM3Dq, P=0.0009;<br>For STN-DBS+mCherry vs. STN-DBS+hM3Dq, P=0.0001.	Two-tailed
Fig. 6J	Number of FS/min	Passed	One-way ANOVA followed by post hoc Tukey's tests	F (3, 20) = 5.026, P=0.0093;<br>For sham STN-DBS vs. STN-DBS, P=0.0342;<br>For STN-DBS vs. STN-DBS+hM3Dq, P=0.0224.	Two-tailed
Fig. 6K	Number of GS/h	Passed	One-way ANOVA followed by post hoc Tukey's tests	F (3, 20) = 8.307, P=0.0009;<br>For sham STN-DBS vs. STN-DBS, P=0.0112;<br>For STN-DBS vs. STN-DBS+hM3Dq, P=0.0021;<br>For STN-DBS+mCherry vs. STN-DBS+hM3Dq, P=0.0148.	Two-tailed
Fig. 6L	Seizure stage	Passed	Two-way repeated ANOVA with Geisser-Greenhouse's correction followed by post hoc Tukey's tests	Interaction: F (45, 300) = 2.295, P<0.0001;<br>Time: F (7.550, 151.0) = 49.92, P<0.0001;<br>Group: F (3, 20) = 13.87, P<0.0001;<br>For sham STN-DBS vs. STN-DBS, P=0.0256;<br>For STN-DBS vs. STN-DBS+hM3Dq, P=0.0126;<br>For STN-DBS+mCherry vs. STN-DBS+hM3Dq, P=0.0218.	Two-tailed
Fig. 7B	Relative concentrations of OA	Passed	One-way ANOVA followed by post hoc Tukey's tests	F (3, 20) = 23.97, P<0.0001;<br>For saline vs. PNC, P<0.0001;<br>For sham vs. STN-DBS, P=0.0013.	Two-tailed
Fig. 7C	Relative concentrations of OB	Passed	One-way ANOVA followed by post hoc Tukey's tests	F (3, 20) = 14.78, P<0.0001;<br>For saline vs. PNC, P=0.0006;<br>For sham vs. STN-DBS, P=0.0013.	Two-tailed
Fig.	Relative	Passed	One-way ANOVA	F (3, 20) = 11.15, P=0.0002;	Two-tailed

7E	protein level of OX1R		followed by post hoc Tukey’s tests	For saline vs. PNC, P=0.0007;<br>For sham vs. STN-DBS, P=0.0485.	
Fig. 7F	Relative protein level of OX2R	Passed	One-way ANOVA followed by post hoc Tukey’s tests	F (3, 20) = 20.41, P<0.0001;<br>For saline vs. PNC, P<0.0001;<br>For sham vs. STN-DBS, P=0.0008.	Two-tailed
Fig. 7J	Spikes/min	Passed	Two-way repeated ANOVA with Geisser-Greenhouse's correction followed by post hoc Tukey’s tests	Interaction: F (45, 300) = 1.001, P=0.4757;<br>Time: F (8.771, 175.4) = 0.9904, P=0.4487;<br>Group: F (3, 20) = 9.873, P=0.0003;<br>For VEH vs. SB-334867, P=0.0329;<br>For VEH vs. SB-334867+JNJ-10397049, P<0.0001.	Two-tailed
Fig. 7K	Spike amplitude (mV)	Passed	Two-way repeated ANOVA with Geisser-Greenhouse's correction followed by post hoc Tukey’s tests	Interaction: F (45, 300) = 1.146, P=0.2519;<br>Time: F (8.872, 177.4) = 0.8514, P=0.5685;<br>Group: F (3, 20) = 11.06, P=0.0002;<br>For VEH vs. SB-334867, P=0.0326;<br>For VEH vs. JNJ-10397049, P=0.0001;<br>For VEH vs. SB-334867+JNJ-10397049, P<0.0001.	Two-tailed
Fig. 7L	EEG power (uV^2)	Passed	Two-way ANOVA followed by post hoc Tukey’s tests	Interaction: F (12, 100) = 1.924, P=0.0400;<br>Band: F (4, 100) = 85.46, P<0.0001;<br>Group: F (3, 100) = 8.551, P<0.0001;<br>For δ, VEH vs. SB-334867+JNJ-10397049, P=0.0018;<br>For β, VEH vs. SB-334867, P=0.0128;<br>For β, VEH vs. JNJ-10397049, P<0.0001;<br>For β, VEH vs. SB-334867+JNJ-10397049, P<0.0001.	Two-tailed
Fig. 7M	Latency to FS (min)	Passed	One-way ANOVA followed by post hoc Tukey’s tests	F (3, 20) = 8.057, P=0.0010;<br>For VEH vs. SB-334867+JNJ-10397049, P=0.0005.	Two-tailed
Fig. 7N	Latency to GS (min)	Passed	One-way ANOVA followed by post hoc Tukey’s tests	F (3, 20) = 4.233, P=0.0180;<br>For VEH vs. SB-334867+JNJ-10397049, P=0.0106.	Two-tailed
Fig. 7O	Number of FS/min	Passed	One-way ANOVA followed by post hoc Tukey’s tests	F (3, 20) = 3.688, P=0.0291;<br>For VEH vs. SB-334867+JNJ-10397049, P=0.0219.	Two-tailed
Fig. 7P	Number of GS/h	Passed	One-way ANOVA followed by post hoc Tukey’s tests	F (3, 20) = 4.258, P=0.0177;<br>For VEH vs. SB-334867+JNJ-10397049, P=0.0133.	Two-tailed
Fig. 7Q	Seizure stage	Passed	Two-way repeated ANOVA with Geisser-Greenhouse's correction followed by post hoc Tukey’s tests	Interaction: F (45, 300) = 1.287, P=0.1144;<br>Time: F (7.847, 156.9) = 25.55, P<0.0001;<br>Group: F (3, 20) = 9.018, P=0.0006;<br>For VEH vs. SB-334867+JNJ-10397049, P=0.0010.	Two-tailed

sFig. 1D	Adjusted number of GS/h	Passed	One-way ANOVA followed by post hoc Tukey's tests	F (2, 15) = 10.47, P=0.0014;<br>For sham vs. ipsi, P=0.0084,<br>For sham vs. bi, P=0.0017.	Two-tailed
sFig. 2E	Number of FS/min	Passed	Paired T tests	For CNO vs. pre, t=3.639, df=5, P=0.0149;<br>For CNO vs. post, t=4.899, df=3, P=0.0163.	Two-tailed
sFig. 2F	Latency to FS (min)	Passed	Paired T tests	For CNO vs. pre, t=5.000, df=5, P=0.0041;<br>For CNO vs. post, t=3.656, df=3, P=0.0354.	Two-tailed
sFig. 2G	Number of GS/h	Passed	Paired T tests	For CNO vs. pre, t=6.220, df=5, P=0.0016;<br>For CNO vs. post, t=3.220, df=3, P=0.0486.	Two-tailed
sFig. 2H	Latency to GS (min)	Passed	Paired T tests	For CNO vs. pre, t=5.918, df=5, P=0.0020;<br>For CNO vs. post, t=2.875, df=3, P=0.0638.	Two-tailed
sFig. 2I	Number of FS/min	Passed	Paired T tests	For CNO vs. pre, t=2.666, df=5, P=0.0446;<br>For CNO vs. post, t=3.124, df=5, P=0.0261.	Two-tailed
sFig. 2J	Latency to FS (min)	Passed	Paired T tests	For CNO vs. pre, t=3.140, df=5, P=0.0257;<br>For CNO vs. post, t=3.162, df=5, P=0.0250.	Two-tailed
sFig. 2K	Number of GS/h	Passed	Paired T tests	For CNO vs. pre, t=3.997, df=5, P=0.0104;<br>For CNO vs. post, t=4.339, df=5, P=0.0074.	Two-tailed
sFig. 2L	Latency to GS (min)	Passed	Paired T tests	For CNO vs. pre, t=4.052, df=5, P=0.0098;<br>For CNO vs. post, t=4.382, df=5, P=0.0071.	Two-tailed
sFig. 3A	Adjusted number of GS/h	Passed	One-way ANOVA followed by post hoc Tukey's tests	F (4, 25) = 11.10, P<0.0001;<br>For mCherry+CNO vs. hM3Dq+CNO, P=0.0011,<br>For hM3Dq+saline vs. hM3Dq+CNO, P=0.0072,<br>For hM4Di+saline vs. hM4Di+CNO, P=0.0258.	Two-tailed
sFig. 3B	Adjusted number of GS/h	Passed	One-way ANOVA followed by post hoc Tukey's tests	F (3, 20) = 10.26, P=0.0003;<br>For sham STN-DBS vs. STN-DBS, P=0.0078,<br>For STN-DBS vs. STN-DBS+hM3Dq, P=0.0007,<br>For STN-DBS+mCherry vs. STN-DBS+hM3Dq, P=0.0040.	Two-tailed
sFig. 3B	Adjusted number of GS/h	Passed	One-way ANOVA followed by post hoc Tukey's tests	F (3, 20) = 5.022, P=0.0093;<br>For VEH vs. SB-334867+JNJ-10397049, P=0.0056.	Two-tailed
